# A Novel Ruthenium-based Molecular Sensor to Detect Endothelial Nitric Oxide

**DOI:** 10.1038/s41598-019-39123-3

**Published:** 2019-02-08

**Authors:** Achini K. Vidanapathirana, Benjamin J. Pullen, Run Zhang, MyNgan Duong, Jarrad M. Goyne, Xiaozhou Zhang, Claudine S. Bonder, Andrew D. Abell, Christina A. Bursill, Stephen J. Nicholls, Peter J. Psaltis

**Affiliations:** 1 Vascular Research Centre, Heart Health Theme, South Australian Health and Medical Research Institute (SAHMRI), Adelaide, South Australia 5000 Australia; 2 Australian Research Council (ARC) Centre of Excellence for Nanoscale BioPhotonics (CNBP), Adelaide, Australia; 3 Australian Institute for Bioengineering and Nanotechnology (AIBN), University of Queensland, St Lucia, Queensland, 4072 Australia; 4 Adelaide Medical School, University of Adelaide, Adelaide, South Australia 5000 Australia; 5 Institute for Photonics and Advanced Sensing (IPAS), School of Physical Sciences. University of Adelaide, Adelaide, South Australia 5000 Australia; 6 Department of Chemistry, University of Adelaide, Adelaide, South Australia 5000 Australia; 7 Centre for Cancer Biology, SA Pathology and University of South Australia, Adelaide South Australia 5000, Australia

## Abstract

Nitric oxide (NO) is a key regulator of endothelial cell and vascular function. The direct measurement of NO is challenging due to its short half-life, and as such surrogate measurements are typically used to approximate its relative concentrations. Here we demonstrate that ruthenium-based [Ru(bpy)_2_(dabpy)]^2+^ is a potent sensor for NO in its irreversible, NO-bound active form, [Ru(bpy)_2_(T-bpy)]^2+^. Using spectrophotometry we established the sensor’s ability to detect and measure soluble NO in a concentration-dependent manner in cell-free media. Endothelial cells cultured with acetylcholine or hydrogen peroxide to induce endogenous NO production showed modest increases of 7.3 ± 7.1% and 36.3 ± 25.0% respectively in fluorescence signal from baseline state, while addition of exogenous NO increased their fluorescence by 5.2-fold. The changes in fluorescence signal were proportionate and comparable against conventional NO assays. Rabbit blood samples immediately exposed to [Ru(bpy)_2_(dabpy)]^2+^ displayed 8-fold higher mean fluorescence, relative to blood without sensor. Approximately 14% of the observed signal was NO/NO adduct-specific. Optimal readings were obtained when sensor was added to freshly collected blood, remaining stable during subsequent freeze-thaw cycles. Clinical studies are now required to test the utility of [Ru(bpy)_2_(dabpy)]^2+^ as a sensor to detect changes in NO from human blood samples in cardiovascular health and disease.

## Introduction

Nitric oxide (NO) is a ubiquitous, gaseous molecule that acts as a messenger in numerous regulatory functions of various cells and tissues^1^. It plays a significant role within the cardiovascular system as a potent vasodilator at lower concentrations (pm-nm range) produced by endothelial nitric oxide synthase (eNOS), alongside well-studied protective mechanisms in early stages of pathological processes such as atherosclerosis and ischaemic heart disease^2,3^. Optimum physiological concentrations of NO are tissue specific^4^ with relatively higher concentrations (µM range) produced by inducible nitric oxide synthase (iNOS) associated with detrimental consequences in inflammation and septic shock. The small size, volatility, short half-life (approximately 2 ms)^5^ and other physical properties of NO present considerable challenges in developing reliable methods for its detection and accurate measurement within blood, cells and tissues. Many fluorescence-based sensors including diaminofluorescein^6,7^, BODIPY^8^, Near Infra-Red fluorescence^9–12^, carbon-nanotube^9,10^ and metal-based turn-on fluorescent probes^13,14^ have been developed to detect NO in cells, tissues and organs^15,16^. Electrochemical methods have been applied for NO sensing, leading to the development of many chemical multimodality sensors that have significant limitations based on their physical and chemical properties and toxicological profiles^17–19^. Some studies have also reported attempts to attach different sensors, including heme domain of guanylate cyclase^20^, cytochrome c^21^ and a gold adsorbed fluorophore^22^ onto fibre-optic probes as potentially translatable approaches that can measure NO *in vivo*. However, these NO sensors have not progressed to clinical applications due to unfavourable characteristics of the sensors, their stability, and interactions with other ligands or blood components.

In the current study, a responsive Ruthenium(II) complex-based luminescence sensor^23^, [Ru(bpy)_2_(dabpy)]^2+^, was employed as the molecular probe for detecting NO. This complex is weakly luminescent due to effective photo-induced electron transfer from *o*-diaminophenyl to Ru(II) centre. Luminescence is switched on as a result of a specific chemical reaction triggered by NO, forming [Ru(bpy)_2_(T-bpy)]^2+^ ^23^. Potential utility of this sensor is based on: a) the unique photo-physical properties of Ru(II) complexes, such as large stokes shift (approximately 165 nm)^23^, long luminescence lifetime, and photostability^24^; (b) fast responsiveness and high specificity towards NO detection in buffer and live systems^23,24^; (c) desirable biocompatibility, which enables NO detection in biological systems^23,24^; (d) pH independent luminescence, allowing NO detection in weakly acidic, neutral, and basic media^23^; and (e) cell membrane permeability, allowing visualisation of intracellular NO^23,24^. As reported previously^23^, the increase in luminescence intensity of this sensor is NO-concentration dependent, making it possible to obtain a relative quantification of NO levels.

This study was designed to assess the applicability of [Ru(bpy)_2_(dabpy)]^2+^ and the NO bound form [Ru(bpy)_2_(T-bpy)]^2+^ to obtain a quantifiable measure of endothelial and plasma derived NO. Here we demonstrate that [Ru(bpy)_2_(dabpy)]^2+^ can be used as a reliable, potent, concentration-dependent NO sensor in cell-free media, endothelial cell culture and rabbit plasma. The sensor is not well internalised or surface-bound in endothelial cells, suggesting utility for the measurement of soluble NO in the circulation and possibly tissue. Specific sensor characteristics are identified, applicable to the future development of photometric test-strip or optic-fibre based probes, potentially allowing the use of this sensor to detect and measure NO in clinical settings.

## Results and Discussion

### [Ru(bpy)_2_(dabpy)]^2+^ as a sensor for NO

The [Ru(bpy)_2_(dabpy)]^2+^ complex displays good selectivity for NO relative to other reactive oxygen and nitrogen species within a µM detection range for NO^23^. The sensor can be switched on upon targeted capturing of NO in buffer solution and in live organisms resulting in [Ru(bpy)_2_(T-bpy)]^2+^ (Fig. S1). These ruthenium-based sensors show fast luminescence responses towards NO with the reaction rate of 10^10^ M^−1^s^−1^, and a detection limit of 2.7 × 10^−7^ M^23,24^. We used fluorescence spectrophotometry to assess the ability of [Ru(bpy)_2_(dabpy)]^2+^ to detect NO in cell-free media, endothelial cell culture and plasma. Based on equipment sensitivity and logistics, the Glomax Discover System (Promega) was used for cell-free background control studies and endogenous agent concentration-based responses in cell culture. A SynergyMx Microplate Reader (BioTek) was used to assess different emission wavelengths, biological replicates of cell culture and rabbit plasma.

### Detecting NO in cell-free media

**Figure 1:**
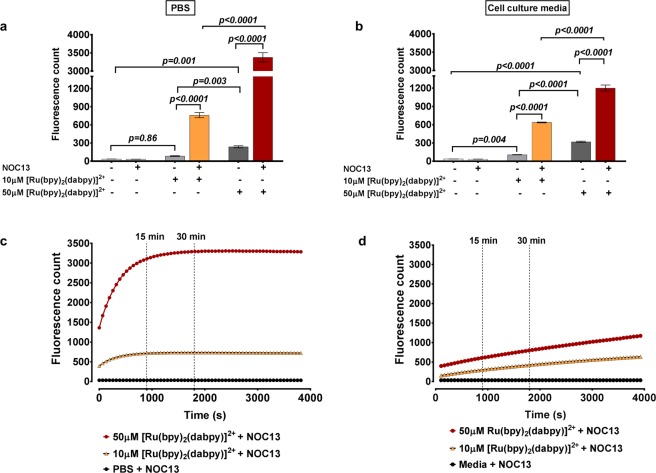
Comparison of fluorescence counts from 10 and 50 µM Ru(bpy)_2_(dabpy)]^2+^ in cell-free PBS and cell culture media. (**a**,**b**) Fluorescence counts under λ_ex_ = 450 nm and λ_em_ = 615 nm on the Glomax Discover System with 10 µM or 50 µM [Ru(bpy)_2_(dabpy)]^2+^, in the absence or presence of the NO donor, NOC13 (1 mM for 60 minutes) in cell-free PBS and phenol red-free M199 cell culture media. The *p-values* were derived from one-way ANOVA followed by Tukey’s multiple comparisons test. (**c**,**d**) Representative fluorescence count readings over 60 minutes under λ_ex_ = 450 nm and λ_em_ = 615 nm after the addition of NOC13 (1 mM) to 10 µM or 50 µM [Ru(bpy)_2_(dabpy)]^2+^ in cell-free PBS and in phenol red-free M199 cell culture media. All data are represented as mean ± s.d. from 3–6 cell-free replicates.

Spectrophotometric readings were compared from 10 and 50 µM [Ru(bpy)_2_(dabpy)]^2+^ to detect NO in cell-free phosphate buffered saline (PBS) or phenol red-free cell culture media in the presence of a NO donor, NOC13 (1-hydroxy-2-oxo-3-(3-amino-propyl)-3-methyl-1-triazene, stoichiometry of NOC13:NO is 1:2 and T_1/2_ = 13.7 min at 22 °C). Ruthenium-based NO sensor concentrations of 10, 50 and 500 µM have been used previously^23,24^ and we chose to test the lower concentrations before *in vitr*o application under our experimental conditions. The 50 µM of [Ru(bpy)_2_(dabpy)]^2+^ produced a larger fluorescence response (compared to 10 µM) to the addition of NOC13 in both PBS and cell culture media (Fig. 1a,b). However, [Ru(bpy)_2_(dabpy)]^2+^ itself generated a concentration-dependent baseline fluorescence, which increased after the addition of NOC13 (Fig. 1c,d). Fluorescence counts were consistent across two batches of working solutions of [Ru(bpy)_2_(dabpy)]^2+^ used for *in vitr*o studies, suggesting that the sensor is stable during storage in suspension (Fig. S2). Following these verifications, 50 µM [Ru(bpy)_2_(dabpy)]^2+^ was selected for further studies, unless specified otherwise. We observed lower baseline fluorescence counts with cell culture medium compared to PBS in the above mentioned cell-free assessments. This difference could be attributed to: (1) the quenching of Ru-products by the constituents of cell culture medium; (2) the slower release of NO by NOC13 in cell culture medium; (3) slower response of [Ru(bpy)_2_(dabpy)]^2+^ to NO in cell culture medium and (4) relatively higher background auto-fluorescence of cell culture medium due to its constituents such as foetal bovine serum, compared to PBS. Therefore, baseline fluorescence counts of the sensor are presented where possible, alongside stimulated/treatment fluorescence counts when presenting the results of all experiments. The ∆ fluorescence between these two conditions was used for comparison.

**Figure 2:**
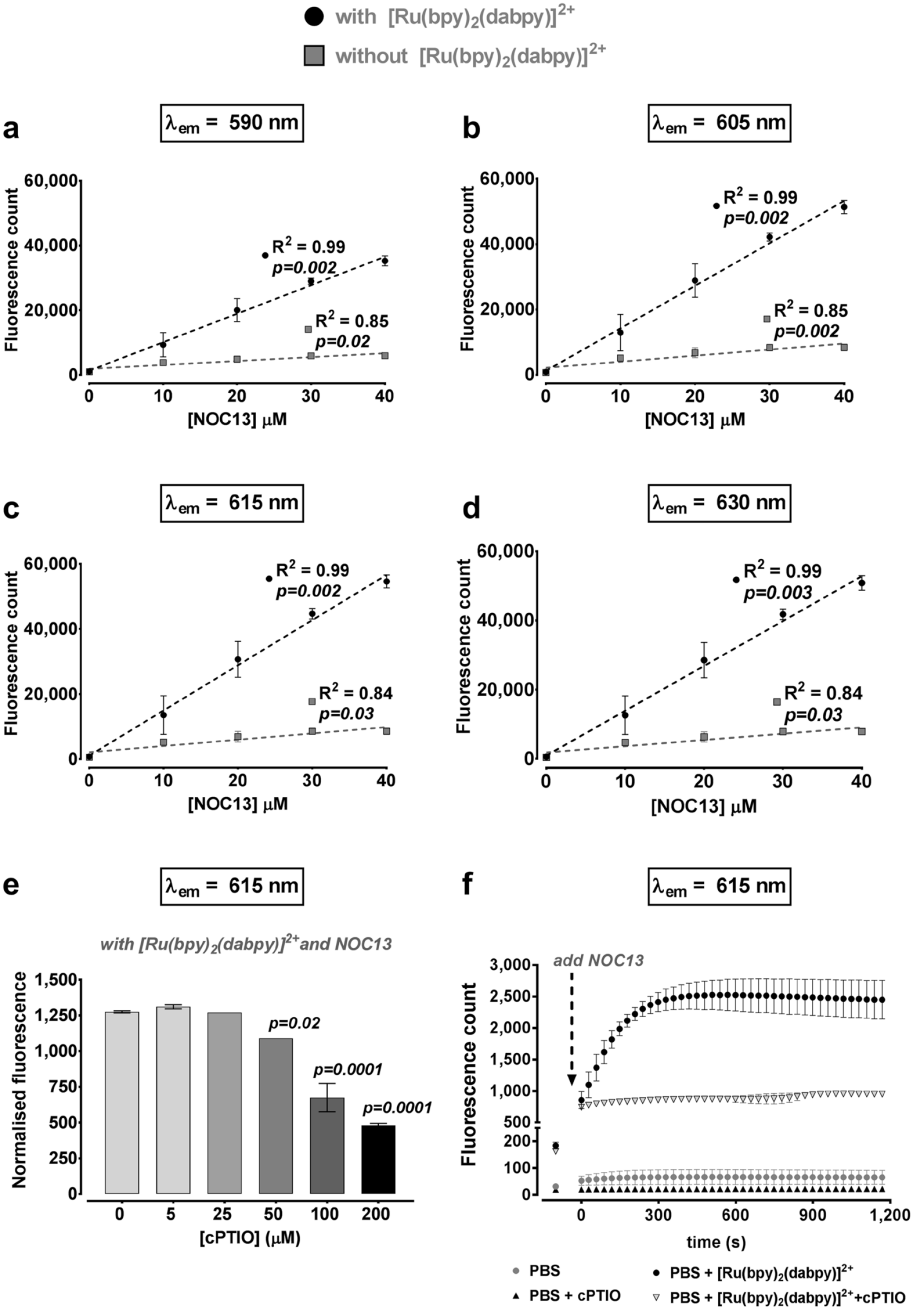
Nitric oxide detection in cell-free media using [Ru(bpy)_2_(dabpy)]^2+^. (**a**–**d**) Fluorescence counts under λ_ex_ = 450 nm and λ_em_ = 590 nm, 605 nm, 615 nm and 630 nm using SynergyMx Microplate Reader, 5 minutes after the addition of the NO donor, NOC13 (10–40 µM) to PBS, with (●) or without () 50 µM [Ru(bpy)_2_(dabpy)]^2+^. The discontinuous lines represent the best fit used for the regression analysis and to calculate the coefficient of determination (R^2^) for each emission wavelength (λ_em_). (**e**) Concentration dependent reduction of the fluorescence count readings under λ_ex_ = 450 nm and λ_em_ = 615 nm at the end of 30 minutes after the addition of NOC13 (1 mM) in the presence of an NO scavenger, cPTIO (5–200 µM) and [Ru(bpy)_2_(dabpy)]^2+^ in phenol red-free cell culture media on the Glomax Discover System. The fluorescence count readings are normalised to the initial reading with cPTIO, before adding NOC13 for each sample. Only *p-values* less than 0.05 are reported, derived from one-way ANOVA followed by Dunnett’s multiple comparisons test to 0 µM cPTIO. (**f**) Fluorescence count readings reported over 20 minutes on the Glomax Discover System to demonstrate the stability of the NO detection signal in PBS in the presence and absence of cPTIO and [Ru(bpy)_2_(dabpy)]^2+^ with excess NOC13. The initial reading was recorded before the addition of NOC13. All data presented as mean ± s.d. from 3–4 cell-free replicates.

A series of spectrophotometry experiments using [Ru(bpy)_2_(dabpy)]^2+^ in cell-free PBS was initially performed to determine optimal emission wavelength, concentration-dependent responsiveness to NO and the irreversibility of NO binding. A linear concentration-dependent fluorescence response to NOC13 was observed within a concentration range of 0–40 µM, after just five minutes of reaction time in PBS and this remained stable over 2 hours, at an excitation wavelength (λ_ex_) of 450 nm and at all four emission wavelengths (λ_em_) tested (590, 605, 615 and 630 nm) (Fig. 2a–d). These responses suggest [Ru(bpy)_2_(T-bpy)]^2+^ could be a suitable sensor for physiologically relevant, lower µM concentrations of NO. Following these observations, λ_ex_ = 450 nm and λ_em_ = 615 nm were chosen for further spectrophotometric assessments in order to minimise the overlap with background auto-fluorescence. The concentration-responsiveness of [Ru(bpy)_2_(dabpy)]^2+^ to NO in cell-free PBS was also shown using a different NO donor with longer half-life, NOC5 (3-(aminopropyl)-1-hydroxy-3-isopropyl-2-oxo-1-triazene, T_1/2_ = 93 min at 22 °C, Fig. S3) and by quenching NO in the presence of NOC13 with an NO scavenger, cPTIO (2-(4-carboxyphenyl)-4, 4, 5, 5-tetramethylimidazoline-1-oxyl-3-oxide) (Fig. 2e). Lower fluorescence counts in PBS were observed with cPTIO compared to a [Ru(bpy)_2_(dabpy)]^2+^ only control, in the absence of NOC13. Fluorescence counts substantially increased after addition of excess NOC13, plateauing after 5 min and remaining stable for at least 20 min of follow-up; such fluorescence response was completely absent in the presence of cPTIO (Fig. 2f). These findings confirmed the specificity of [Ru(bpy)_2_(dabpy)]^2+^ to NO and its ability to produce a stable, irreversible response, saturating the sensor capacity as early as 15 min after the addition of excess exogenous NO in PBS.

Prior to the cell-based studies, fluorescence spectrophotometry was used to elucidate any interactions of [Ru(bpy)_2_(dabpy)]^2+^ with agents (acetylcholine and hydrogen peroxide) that were used to stimulate endogenous NO production in endothelial cells. Unlike NOC13, addition of 10 µM acetylcholine or 150 µM hydrogen peroxide (H_2_O_2_) to cell-free PBS or cell culture media resulted in negligible changes in [Ru(bpy)_2_(T-bpy)]^2+^ fluorescence count. This is consistent with the fact that these are stimuli of cellular release for NO and have minimal or no interference with the [Ru(bpy)_2_(T-bpy)]^2+^ fluorescence counts (Figs S4 and S5). Interestingly, fluorescence counts were observed to be relatively lower when NOC13 was added to culture media but steadily increased over time; in contrast, fluorescence counts in PBS were higher but consistently plateaued after approximately 5 min (see Figs 1c,d, S2, S4 and S5). These differences are likely due to the inherent presence of NO producing or scavenging essential constituents in cell culture media^25^ that are absent in PBS, but can affect the NOC13 degradation and the availability of NO to bind to [Ru(bpy)_2_(dabpy)]^2+^.

**Figure 3:**
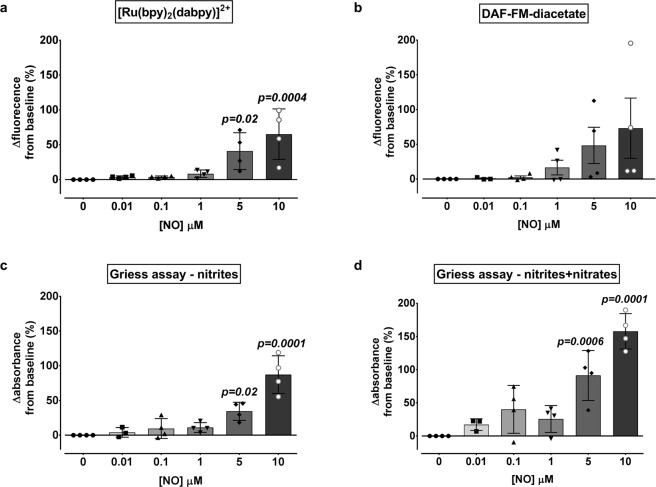
Comparison of sensitivity of [Ru(bpy)_2_(dabpy)]^2+^ with direct and indirect conventional NO assays in cell-free media. Nitric oxide concentration dependent changes in the ∆fluorescence calculated as a percentage from the baseline reading in the absence of NO (0 µM NOC13) for (**a**) [Ru(bpy)_2_(dabpy)]^2+^ at λ_ex_ = 450 nm and λ_em_ = 615 nm and for (**b**) DAF-FM-diacetate at λ_ex_ = 490 nm and λ_em_ = 515 nm. Comparable changes in the ∆absorbance at λ_ab_ = 560 nm calculated as a percentage from the baseline reading in the absence of NO, derived from the Griess assay for (**c**) nitrites only and (**d**) total nitrites + nitrates. Each bar represents the mean percentage ± s.d of four independent experiments and the symbols represent the mean percentage of 2–3 replicates in each experiment in cell free PBS, using the same spectrophotometer (Glomax Discover System, Promega). The NOC13 concentrations were adjusted to account for the relevant time points in each assay and to compare the response at each concentration of NO. Only *p-values* less than 0.05 are reported, based on one-way ANOVA followed by Dunnett’s multiple comparisons test to 0 µM NO.

We also compared the NO sensitivity of [Ru(bpy)_2_(dabpy)]^2+^ in cell-free PBS to two more conventional measures of NO using spectrophotometry. The 10 µM [Ru(bpy)_2_(dabpy)]^2+^ demonstrated significantly higher changes in fluorescence counts acquired only above 1 µM NO (Fig. 3a). These changes were comparable against the direct sensor 10 µM DAF-FM-diacetate (4-Amino-5-methylamino-2′, 7′-difluorofluorescein diacetate) (Fig. 3b). When compared to both nitrite only and nitrite + nitrate readings in the indirect NO sensing Griess assay, [Ru(bpy)_2_(dabpy)]^2+^ generated comparable responses at each incremental concentration (Fig. 3c,d). All direct and indirect sensors/assays were minimally sensitive to changes below 1 µM NO and could also potentially detect some NO metabolites such as NO^+^ in solution. The specificity of [Ru(bpy)_2_(dabpy)]^2+^ to NO in comparison to other NO metabolites such as NO_2_^−^, NO_3_^−^ and ONOO^−^ has already been demonstrated^23^. Considering the percentage changes of fluorescence and extrapolating on actual amounts of NO detected, based on NOC13 concentration used in our study with the stoichiometry, we can conclude that the detection threshold of [Ru(bpy)_2_(dabpy)]^2+^ is similar to DAF-FM-diacetate and the Griess assay. We elected to use DAF-FM-diacetate, instead of the Griess assay, for further comparisons with [Ru(bpy)_2_(dabpy)]^2+^ in subsequent cell-based studies, as it directly senses NO and requires a similar time-frame for generating the ∆fluorescence response.

The micromolar range of NO detection reported in the current study with [Ru(bpy)_2_(dabpy)]^2+^ is biologically relevant, particularly at the lower micromolar concentrations. However, we could not demonstrate its sensitivity within the nanomolar range in our *in vitro* studies. Recent attempts with a microplate reader using DAF-FM reported detection of NO concentrations below 10 nM^26^, which could not be replicated under our experimental conditions with either DAF-FM-diacetate or [Ru(bpy)_2_(dabpy)]^2+^.

### [Ru(bpy)_2_(dabpy)]^2+^ does not affect the viability and function of HUVECs

**Figure 4:**
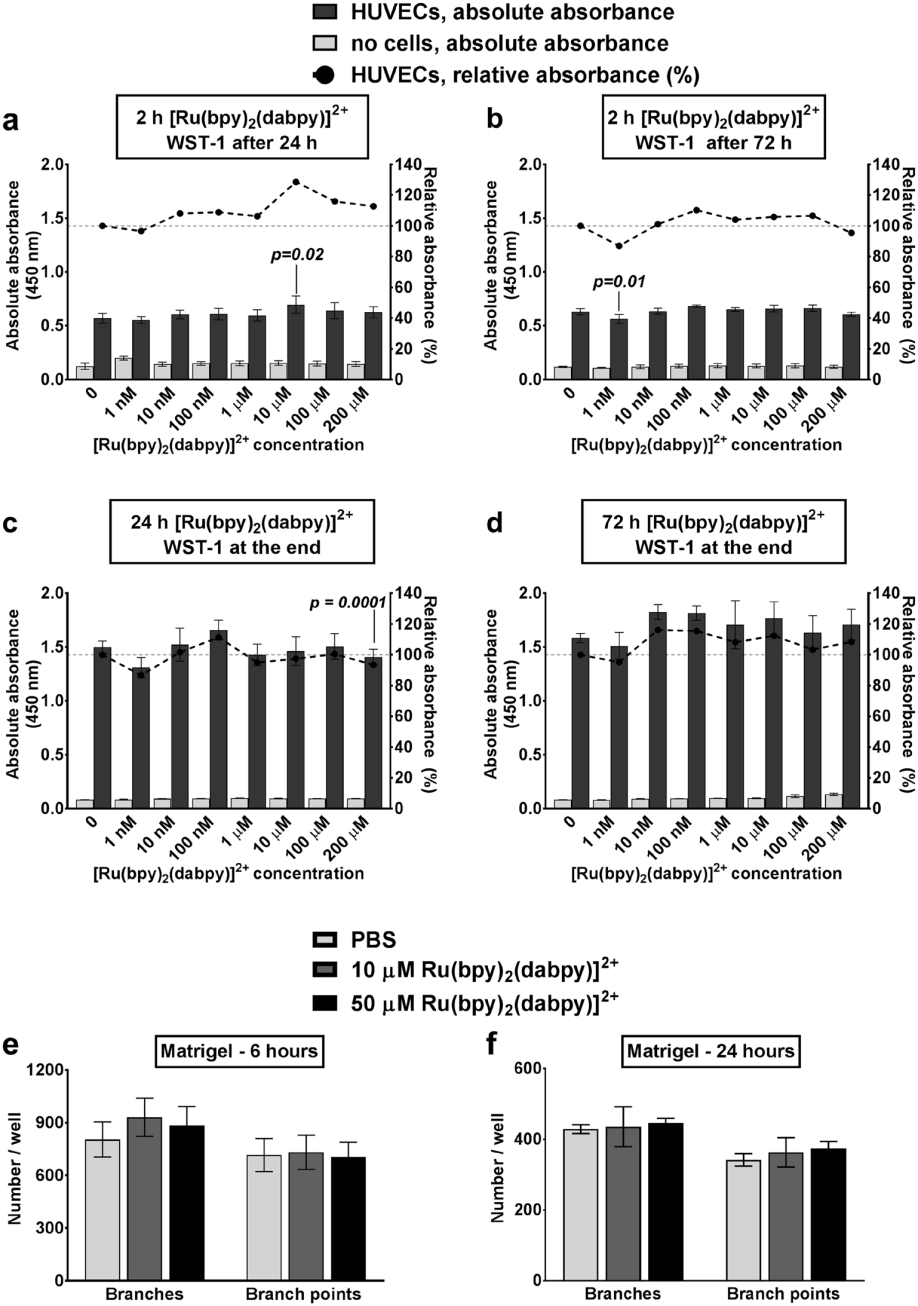
Viability and function of HUVECs in the presence of [Ru(bpy)_2_(dabpy)]^2+^. Absorbance at 450 nm with WST-1 reagent for HUVECs exposed to 1 nM –200 µM [Ru(bpy)_2_(dabpy)]^2+^ (mean ± s.d. from quadruplicate experiment) at four time points of exposure. Relative absorbance is expressed as a percentage in the right Y axis (––), considering cell absorbance without any [Ru(bpy)_2_(dabpy)]^2+^ as 100% (). The changes in cell viability at (**a**) 2 h with [Ru(bpy)_2_(dabpy)]^2+^, washed with culture media and WST-1 assay after 24 h, (**b**) 2 h with [Ru(bpy)_2_(dabpy)]^2+^, washed with culture media and WST-1 assay after 72 h, (**c**) 24 h with [Ru(bpy)_2_(dabpy)]^2+^, WST-1 assay at the end of exposure, and (**d**) 72 h with [Ru(bpy)_2_(dabpy)]^2+^, WST-1 assay at the end of exposure are reported. HUVECs were seeded at 4000 cells/well for all conditions and incubated at 37 °C and 5% CO_2_ throughout the assay. Only *p-values* less than 0.05 are reported, based on one-way ANOVA followed by Dunnett’s multiple comparisons test to 0 nM [Ru(bpy)_2_(dabpy)]^2+^. Number of branches and branch points per well (mean ± s.d. from quadruplicate experiment) formed on Matrigel at (**e**) 6 hours and (**f**) 24 hours of vascular tube formation following 24 hours pre-exposure to 10 or 50 µM [Ru(bpy)_2_(dabpy)]^2+^. Following pre-exposure, the HUVECs were seeded at 1.2 × 10^4^ cells/well for all conditions and incubated at 37 °C and 5% CO_2_ throughout the assay. The differences between groups were not significant based on one-way ANOVA followed by Tukey’s multiple comparisons test (Representative images for each condition are included in Fig. S8).

Human Umbilical Vein Endothelial Cells (HUVECs) were used to test the applicability of [Ru(bpy)_2_(dabpy)]^2+^ as a sensor to detect endogenous and exogenous NO in the context of primary human vascular endothelial cells. The Water Soluble Tetrazolium-1 (WST-1) assay was first used to test the toxicity of [Ru(bpy)_2_(dabpy)]^2+^ on HUVECs *in vitro*, as a basic biocompatibility assessment across a concentration range of 1 nM to 200 µM, and an exposure duration of 2 (Fig. 4a,b), 24 (Fig. 4c) and 72 (Fig. 4d) hours. Despite the presence of very minor changes in cell viability, all concentrations and exposure durations resulted in WST-1 absorbance values of >86% when compared to HUVECs cultured in the absence of [Ru(bpy)_2_(dabpy)]^2+^. As verified using [Ru(bpy)_2_(dabpy)]^2+^-only cell-free controls, [Ru(bpy)_2_(dabpy)]^2+^ has minimal interference with WST-1 reagent and its absorbance remains stable over 72 hours. In addition, we did not observe significant changes in well-studied endothelial cell functions such as vascular tube forming capacity (Figs 4e,f and S8) or an increase in the general oxidative stress in response to [Ru(bpy)_2_(dabpy)]^2+^ exposure (Fig. S9). These observations indicated that Ru(bpy)_2_(dabpy)]^2+^ or its NO bound active form [Ru(bpy)_2_(T-bpy)]^2+^ did not adversely affect HUVEC viability, proliferation or function. This is the first time *in vitro* toxicity studies have been performed with Ruthenium-based molecules or nanoparticles with primary human endothelial cells. However, [Ru(bpy)_2_(dabpy)]^2+^ being of sub-nanoscale size, could result in variable toxicity profiles at higher/lower concentrations based on zeta potential, particle size in solution, agglomeration properties, biomolecular interactions and generation of reactive oxygen species as reported with other metal-based nanoparticles^27,28^. Therefore these cytotoxicity data reflecting minimal toxic effects on endothelial cells need to be reinforced by further assessments of sensor distribution, uptake and clearance, before progressing to any *in vivo* or clinical applications of this sensor.

### Detecting endogenous and exogenous NO in HUVECs in culture

**Figure 5:**
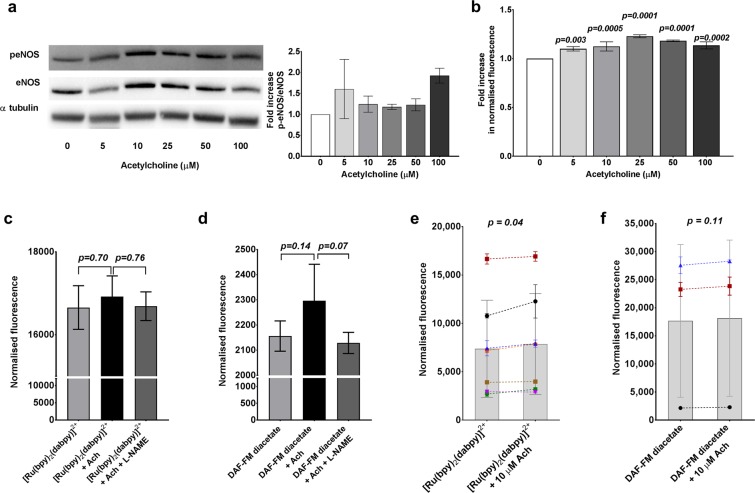
Detection of changes in endogenous NO in the presence of acetylcholine in HUVECs. (**a**) Acetylcholine (Ach) stimulates endogenous production of NO by phosphorylation of eNOS as demonstrated by the representative Western blot protein analysis for p-eNOS, eNOS and αtubulin, in HUVECs treated with 5–100 µM Ach, (15 mins at 37 °C and 5% CO_2_). (Full-length blots, exposure time and details of antibodies are included in Fig. S14). The bar graph represents the fold increase in p-eNOS/eNOS ratio normalised to 0 µM Ach, derived from the densitometry of protein bands in two experiments done with different HUVEC donors. (**b**) [Ru(bpy)_2_(dabpy)]^2+^ was able to directly detect corresponding changes in NO as represented by the concentration dependent changes in fluorescence from triplicate readings from HUVECs on the Glomax Discover System with 5–100 µM Ach (15 mins, at 37 °C and 5% CO_2_) and 50 µM [Ru(bpy)_2_(dabpy)]^2+^. The *p-values* were derived from one-way ANOVA followed by Dunnett’s multiple comparisons test to 0 µM Ach. The fluorescence counts (quadruplicate readings) normalised to cell-only controls increased with both (**c**) [Ru(bpy)_2_(dabpy)]^2+^ and (**d**) DAF-FM-diacetate with the addition of 10 µM Ach (15 min) to HUVECs_,_ which decreased with 500 µM L-NAME (30 min), an inhibitor of eNOS. In c and d, the p-values were derived from one-way ANOVA followed by Tukey’s multiple comparisons test. (**e**) A small but significant increase in fluorescence counts was seen with the addition of 10 µM Ach (15 min) in the presence of [Ru(bpy)_2_(dabpy)]^2+^ in HUVECs on SynergyMx Microplate Reader (n = 7). (**f**) Comparable changes in the fluorescence counts with the conventional NO sensor DAF-FM-diacetate and HUVECs, using the same spectrophotometer (n = 3). In graphs e and f, the bars represent the mean fluorescence counts of multiple independent experiments done with HUVECs from different passages/donors. Each symbol represents the fluorescence counts from 3–4 replicates in each donor experiment, normalised to the cells only controls and the discontinuous lines represent the increase in fluorescence count with the endogenous NO stimulation by Ach. The *p-values* were derived from a paired *t-test* between Ach stimulated group and the sensor control. All data are presented as mean ± s.d.

We tested the capacity of [Ru(bpy)_2_(dabpy)]^2+^ to detect NO in cultured, attached endothelial cells in the presence of both endogenous (acetylcholine and H_2_O_2_) and exogenous (NOC13) sources of NO. While 10 µM acetylcholine and 150 µM H_2_O_2_ have been specifically used in previous studies to demonstrate the function of other NO sensors^13,29,30^, both agents have been shown to produce endogenous NO in endothelial cells at different concentrations and incubation periods. We therefore performed Western blot protein quantifications of phosphorylated-endothelial nitric oxide synthase (p-eNOS), the activated eNOS enzyme, with 5–100 µM acetylcholine stimulation in HUVECs to confirm the endogenous activity under our experimental conditions (Fig. 5a). The same conditions were repeated in the presence of [Ru(bpy)_2_(dabpy)]^2+^ and read using a spectrophotometer to detect NO-bound-[Ru(bpy)_2_(T-bpy)]^2+^. Acetylcholine was associated with a concentration-dependent, modest increase in fluorescence signal, ranging from a mean of 10.0 to 22.8% above signal from unstimulated HUVECs (Fig. 5b). The fluorescent count with [Ru(bpy)_2_(dabpy)]^2+^ showed a non-significant trend towards being reduced after incubation with L-NAME (Nω-Nitro-L-arginine methyl ester), an inhibitor for eNOS (Fig. 5c). A similar trend was also observed with DAF-FM-diacetate (Fig. 5d). Addition of 10 µM acetylcholine resulted in a small but significant 7.3 ± 7.1% increase in the mean fluorescence count from baseline in independent experiments using different donors/passages of HUVECs (*p* = *0.04*, paired *t-*test, n = 7) (Fig. 5e). In corresponding experiments, the relative increase in fluorescence seen with DAF-FM-diacetate was 3.9 ± 2.3% (*p* = *0.11*, paired *t-*test, n = 3) (Fig. 5f). Comparing the magnitude of these changes in fluorescence counts between the two direct NO sensors studied, we observed that [Ru(bpy)_2_(dabpy)]^2+^ was comparable with DAF-FM-diacetate, and sensitive to detect minor endogenous changes in NO.

**Figure 6:**
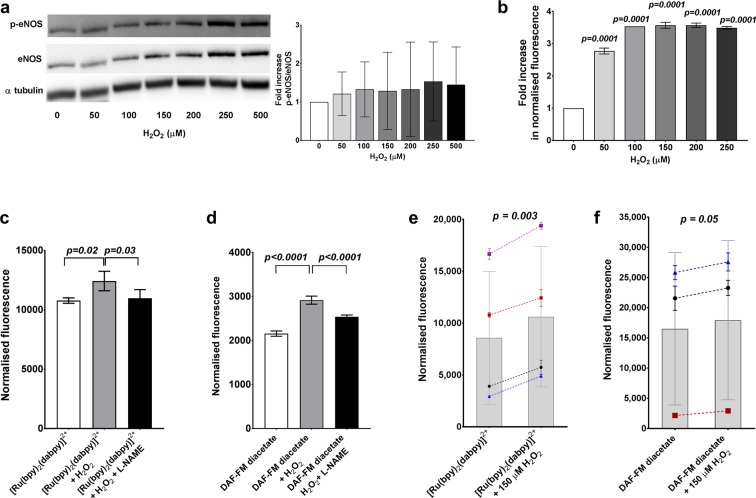
Detection of changes in endogenous NO in the presence of hydrogen peroxide in HUVECs. (**a**) Hydrogen peroxide (H_2_O_2_) stimulates endogenous production of NO by phosphorylation of eNOS as demonstrated by the representative Western blot protein analysis for p-eNOS, eNOS and αtubulin, in HUVECs treated with 5–500 µM H_2_O_2_ (5 min, at 37 °C and 5% CO_2_) (The full-length blots, exposure time and the details of the antibodies are included in Fig. S15). The bar graph represents the fold increase in p-eNOS/eNOS ratio normalised to 0 µM H_2_O_2_ derived from the densitometry of the protein bands in four experiments done with different HUVEC donors/passages. (**b**) [Ru(bpy)_2_(dabpy)]^2+^ was able to directly detect corresponding changes in NO as represented by the concentration dependent changes in fluorescence from triplicate readings on the Glomax Discover System with 50–250 µM H_2_O_2_ (5 min) and 50 µM [Ru(bpy)_2_(dabpy)]^2+^. The *p-values* were derived from one-way ANOVA followed by Dunnett’s multiple comparisons test to 0 µM H_2_O_2._ (**c**) The fluorescence counts increased in both (**c**) [Ru(bpy)_2_(dabpy)]^2+^ and (**d**) DAF-FM-diacetate with the addition of 200 µM H_2_O_2_ (15 min), which decreased significantly in the presence 500 µM L-NAME (30 min), an inhibitor of eNOS. In c and d, the p-values were derived from one-way ANOVA followed by Tukey’s multiple comparisons test between the groups. (**e**) A consistent increase in the fluorescence counts was seen with the addition of 150 µM H_2_O_2_ (15 min) in the presence of [Ru(bpy)_2_(dabpy)]^2+^ in HUVECs on SynergyMx Microplate Reader (n = 4). (**f**) Comparable changes in the fluorescence counts with the conventional NO sensor DAF-FM-diacetate and HUVECs using the same spectrophotometer (n = 3). In graphs e and f, the bars represent the mean fluorescence counts of multiple independent experiments done with HUVECs from different passages/donors. Each symbol represents the fluorescence counts from 3–4 replicates in each donor experiment, normalised to the cells only controls and the discontinuous lines represent the increase in fluorescence with the endogenous NO stimulation by H_2_O_2_. The *p-values* were derived from a paired *t-test* between the H_2_O_2_ stimulated group and the sensor control. All data are presented as mean ± s.d.

Hydrogen peroxide (H_2_O_2_) was used as an additional endogenous stimulus in HUVECs to produce NO and to demonstrate a differential response with the NO sensors. The p-eNOS mediated endogenous activity was confirmed using Western blot protein quantifications with 0–500 µM H_2_O_2_ stimulation in HUVECs (Fig. 6a). We repeated the 0–250 µM H_2_O_2_ conditions with HUVECs in the presence of [Ru(bpy)_2_(dabpy)]^2+^ and read using a spectrophotometer, which resulted in concentration-dependent increases in fluorescence counts ranging from a mean of 175.4 to 254.4% above unstimulated cells (Fig. 6b). These **∆**fluorescence values were markedly higher than those seen with the acetylcholine mediated response. However, this response was not observed in the absence of cells in both PBS and cell culture media (Fig. S6), suggesting minimal interaction between the sensor and H_2_O_2._ The fluorescent counts with [Ru(bpy)_2_(dabpy)]^2+^ decreased significantly after incubation with L-NAME (Fig. 6c) as did the signal seen with DAF-FM-diacetate (Fig. 6d). Addition of 150 µM H_2_O_2_ resulted in a more modest 36.3 ± 25.0% increase in florescence count compared to untreated control in additional independent experiments using different donors/passages of HUVECs (*p* = *0.003*, paired *t-*test n = 4) (Fig. 6e). The same exposure of HUVECs to H_2_O_2_ resulted in a smaller increase of 16.7 ± 16.1% in fluorescence signal above control, using the direct NO sensor DAF-FM-diacetate (*p* = *0.05*, paired *t-*test, n = 3) (Fig. 6f).

**Figure 7:**
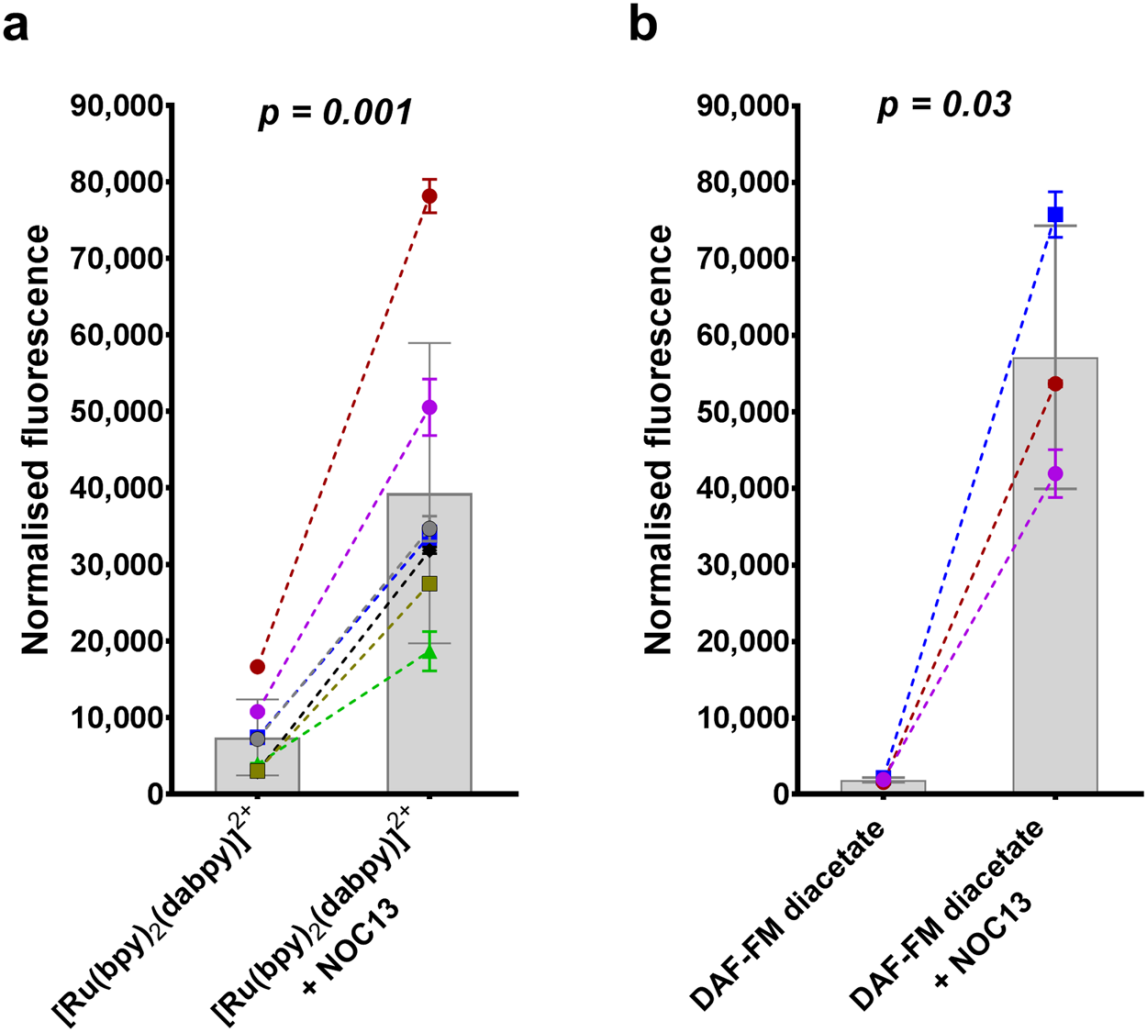
Detection of changes in exogenous NO in the presence of NOC13. (**a**) A significant and consistent increase in the fluorescence counts was observed with the addition of 1 mM NOC13 (30 min at 37 °C and 5% CO_2_), an exogenous NO donor in the presence of 50 µM [Ru(bpy)_2_(dabpy)]^2+^ in HUVECs, on the SynergyMx Microplate Reader (n = 7). (**b**) Marked changes in fluorescence counts were also observed using the conventional NO sensor 10 µM DAF-FM-diacetate with the addition of 1 mM NOC13 (30 min at 37 °C and 5% CO_2_), in HUVECs using the same plate reader (n = 3). In graphs a and b, the bars represent the mean fluorescence counts of multiple independent experiments done with HUVECs from different passages/donors. Each symbol represents the fluorescence counts from 3–4 replicates in each donor experiment, normalised to the cells only controls and the discontinuous lines represent the increase in fluorescence with the addition of exogenous NO with NOC13. The *p-values* were derived from a paired *t-test* between the NOC13 added group and the sensor control. All data are presented as mean ± s.d.

HUVECs were then incubated with excess NOC13 to demonstrate the exogenous NO detection ability of [Ru(bpy)_2_(dabpy)]^2+^ with endothelial cells. Addition of NOC13 resulted in a marked 521.2 ± 262.2% increase in normalised fluorescence count with the Ruthenium sensor (*p* = *0.001* compared to no NOC13, paired *t-*test, n = 7) (Fig. 7a), and a 2944.0 ± 747.6% increase in fluorescence with DAF-FM-diacetate (*p* = *0.03*, paired *t-*test, n = 3) (Fig. 7b).

In the current study, we have demonstrated changes in fluorescence that are similar in magnitude for [Ru(bpy)_2_(dabpy)]^2+^ and DAF-FM diacetate in HUVECs under comparable stimuli. In addition, we also used the Griess assay as an indirect measure of NO under the same endogenous and exogenous conditions. Notably, we did not observe changes in absorbance with the Griess assay in HUVECs under our endogenous experimental conditions in multiple experimental attempts. In contrast, the Griess assay was able to detect changes in absorbance in the presence of the exogenous NO donor NOC13, and these were comparable in magnitude to the other two direct NO sensors (Fig. S7). These observations suggest that the Griess assay is not sensitive enough to detect small, endogenous changes in NO in endothelial cells as it incorporates an indirect/surrogate mechanism to detect NO^31,32^. Differences between the ability to detect NO signal in our study compared to previous studies on HUVECs^29,30,33–35^ could be due to differences in the type, donor source and passage of endothelial cells, stimuli used to induce NO, sensor characteristics and the sensitivity of the equipment used to quantify the fluorescence signal. However, red luminescence emission, large Stokes shift, and long lifetime of this Ru(II) complex sensor render [Ru(bpy)_2_(dabpy)]^2+^ more favourable than DAF–FM diacetate or Griess assay to measure NO in a biological system. NO detection limits of [Ru(bpy)_2_(dabpy)]^2+^ in solution in the current study (1 µM) may be too high for identification of minor changes in levels of NO inside blood vessels. Nevertheless, we observed 7.3% mean increase in fluorescence count with acetylcholine and 36.3% mean increase with H_2_O_2_ induced endogenous NO production, along with a 521.2% mean increase with NOC13, adding excess exogenous NO. These changes in fluorescence are proportionate to the NO availability, with exogenous sources producing markedly higher readings compared to endogenous stimuli. These observations on the sensitivity limits of [Ru(bpy)_2_(dabpy)]^2+^ with cultured endothelial cells support it as a good candidate sensor to be used in early stages of NO probe development.

Nitric oxide production capacity varies with endothelial cell type; nevertheless primary HUVECs are a well-established model to study endothelial cell biology. Acetylcholine and H_2_O_2_ induced NO production via phosphorylation of eNOS are reported in many endothelial NO based studies^13,29,36–38^ including in studies with other NO sensors^13,30^. Hence we chose to treat HUVECs with these endogenous stimuli to demonstrate the applicability of [Ru(bpy)_2_(dabpy)]^2+^ as a NO sensor in endothelial cell culture. However, sensor uptake and the magnitude of change in NO signal that we observed for HUVECs in this study were not as robust as expected, compared to previous reports of other endothelial cell, vascular tissue or organ-based sensor studies^9,13^. Human, porcine or bovine coronary or aortic endothelial cells or live vessel preparations under fluorescence imaging can be used as alternatives to HUVECs to quantitatively demonstrate relatively larger changes in NO levels that could be measured using [Ru(bpy)_2_(dabpy)]^2+^. Additionally, macrophages could be used to test the sensor’s capacity to detect intracellular NO in inflammation, based on their iNOS-mediated production of NO, and phagocytic properties.

### [Ru(bpy)_2_(dabpy)]^2+^ produces a predominantly extracellular signal with HUVECs

We conducted basic spectrophotometric assessments to determine the intracellular versus extracellular localisation of [Ru(bpy)_2_(dabpy)]^2+^ in endothelial cells. Initially, we tested how fluorescence counts were affected by performing media exchanges after HUVECs had been incubated with [Ru(bpy)_2_(dabpy)]^2+^ and treated with acetylcholine or excess NOC13. A substantial loss of fluorescence was observed after changing cell culture media for both stimulatory conditions, and this was recovered in the supernatant collected during the media change (Fig. S10). Together, the different fluorescence intensities observed between cell culture media changes and from the supernatant suggested that much of the fluorescence signal from the [Ru(bpy)_2_(T-bpy)]^2+^ was not intracellular, but predominantly from the cell surface and/or cell media (supernatant). We confirmed these observations by imaging the cells under confocal microscopy in the presence of a nuclear stain, Hoescht, and [Ru(bpy)_2_(dabpy)]^2+^ after addition of NOC13. A substantial reduction of the NOC13 concentration-dependent normalised fluorescence was observed after changing media (Figs S11 and S12). Detailed analysis using inductively coupled plasma mass spectrometry (ICP-MS) confirmed the negligible intake of 0.03% (*p* < *0.05*) particles per billion of Ruthenium by HUVECs, compared to the supernatant (Table S1). These observations differ from previous reports suggesting [Ru(bpy)_2_(T-bpy)]^2+^ being an intracellular sensor in macrophages^23^. This could be explained by greater phagocytic uptake of the sensor by macrophages, along with greater intracellular production of NO by iNOS, the major enzyme responsible for NO synthesis in macrophages.

### NO in rabbit blood

**Figure 8:**
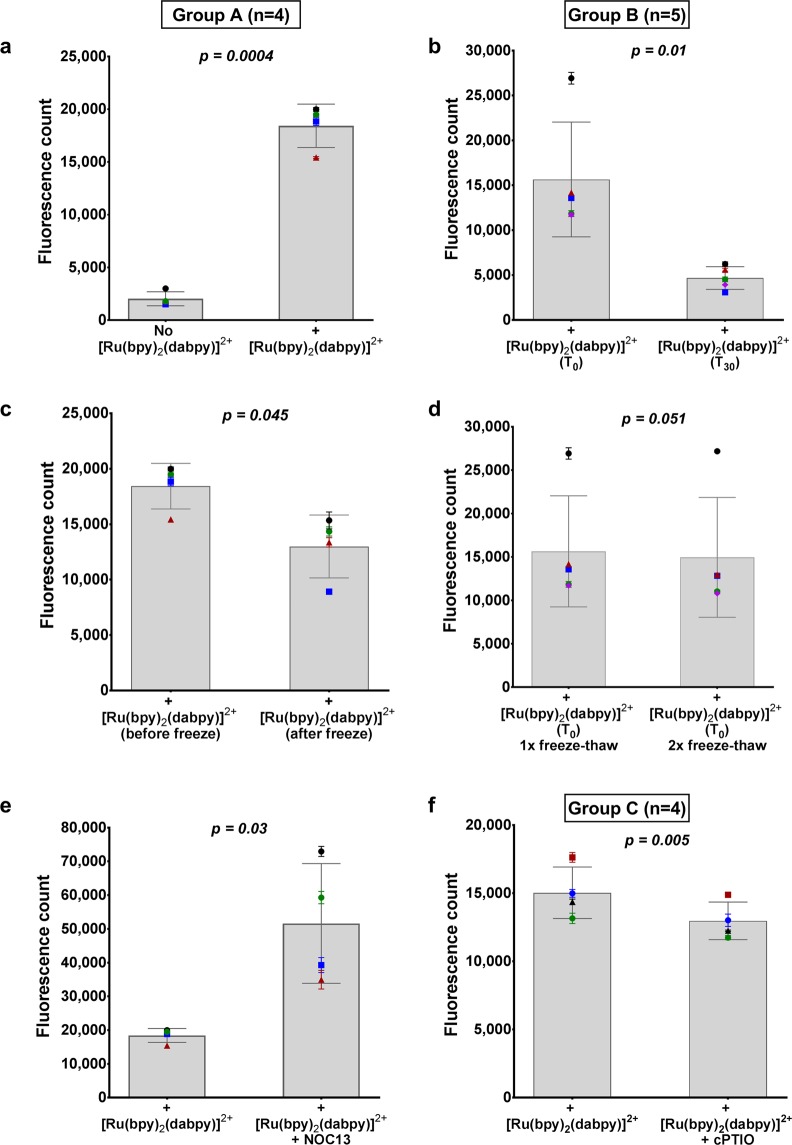
Detection of NO levels in rabbit blood using [Ru(bpy)_2_(dabpy)]^2+^. Fluorescence count readings under λ_ex_ = 450 nm and λ_em_ = 615 nm were taken using a spectrophotometer (SynergyMx Microplate Reader) from plasma samples from blood taken from New Zealand White rabbits in the presence of [Ru(bpy)_2_(dabpy)]^2+^ under different processing conditions and time points (Group A, B and C). (**a**) Comparison of mean fluorescence counts between plasma samples obtained from blood incubated with 50 µM [Ru(bpy)_2_(dabpy)]^2+^ and from blood added to the PBS vehicle control, without [Ru(bpy)_2_(dabpy)]^2+^ in the syringe at the time of collection. (**b**) Comparison of fluorescence counts between plasma from blood incubated with [Ru(bpy)_2_(dabpy)]^2+^ at the time of collection (T_0_) or plasma from blood to which [Ru(bpy)_2_(dabpy)]^2+^ was added after 20 minutes on ice and then centrifuged after a further 10 minutes (T_30_). (**c**) Comparison of fluorescence counts of plasma from blood to which [Ru(bpy)_2_(dabpy)]^2+^ was added at time of collection or to thawed plasma after snap-freezing. (**d**) Comparison of fluorescence counts between 1 and 2 freeze-thaw cycles in plasma from blood incubated with [Ru(bpy)_2_(dabpy)]^2+^ at the time of collection to demonstrate the stability of [Ru(bpy)_2_(T-bpy)]^2+^ during plasma sample processing. (**e**) Demonstration of the presence of active [Ru(bpy)_2_(dabpy)]^2+^ in plasma after a freeze-thaw cycle by adding NOC13 to thawed plasma. (**f**) The control experiments in the presence of [Ru(bpy)_2_(dabpy)]^2+^ with or without cPTIO, a NO scavenger added to blood at the time of collection, snap-frozen, thawed and plasma read under λ_ex_ = 450 nm and λ_em_ = 615 nm. All bar graphs represent the calculated mean ± s.d. in each group and the symbols represent the mean fluorescence from 2–4 replicates from each rabbit. The *p-values* derived from a paired *t-test* between the relevant groups (n = 4–5 rabbits). For further clarification of methods and sample processing, please refer to the schemes in Figs S16 (for a, c and e), S17 (for b and d) and S18 (for f) included under Supplementary Section 4.

Extracellular NO detection capacity of [Ru(bpy)_2_(dabpy)]^2+^ was applied to the detection of NO in rabbit blood using a spectrophotometer, under different sample processing conditions (Figs S16–18). In the first set of experiments, rabbit blood was drawn through cardiac puncture directly to a syringe containing either 50 µM Ru(bpy)_2_(dabpy)]^2+^ or PBS (as vehicle control without Ru(bpy)_2_(dabpy)]^2+^) at time of collection. These samples were then centrifuged, plasma extracted, snap-frozen 30 minutes after collection, stored in −80 °C and thawed prior to reading. We observed an 8-fold higher mean fluorescence count in the plasma derived from [Ru(bpy)_2_(dabpy)]^2+^ containing blood compared to the control without [Ru(bpy)_2_(dabpy)]^2+^ (*p* = *0.0004*, paired *t-*test) (Fig. 8a), suggesting a minimal contribution of the biological fluid (blood/plasma) to the basal non-specific fluorescence readings. Despite the fact that we used consistent blood volumes and a fixed sensor concentration, differences in ∆fluorescence values between [Ru(bpy)_2_(dabpy)]^2+^ and sensor-free control were evident between samples from individual rabbits, which could be due to experimental and/or biological variation. In the second experiment, addition of Ru(bpy)_2_(dabpy)]^2+^ to blood 20 minutes after collection and centrifugation after 10 minutes (T_30_) resulted in a 70.2% lower mean fluorescence count compared to the blood sample added with Ru(bpy)_2_(dabpy)]^2+^ at the time of collection (T_0_) (*p* = *0.01*, paired *t-*test) (Fig. 8b). In addition, a mean 27.5% reduction in fluorescence count was observed when Ru(bpy)_2_(dabpy)]^2+^ was added to the thawed plasma samples after snap-freezing, in comparison to adding Ru(bpy)_2_(dabpy)]^2+^ to blood at collection (*p* = *0.045*, paired *t-*test) (Fig. 8c). Such differences in fluorescence could be due to metabolism of soluble NO in the samples, effects of temperature changes during processing and also variations in the quenching of sensor by different cells in the blood sample that were not included in the analysis. These observations suggest that the application of [Ru(bpy)_2_(dabpy)]^2+^ as a sensor requires the addition of the sensor immediately during collection to the blood sample prior to any reaction limiting interventions such as snap-freezing. Importantly, once the sensor was added to blood samples, fluorescence counts were relatively unaffected in plasma by snap-freezing and one or two freeze-thaw cycles (Fig. 8d), confirming the stability of [Ru(bpy)_2_(T-bpy)]^2+^ in plasma during freeze-thaw processing. This is a positive characteristic for a sensor with a potential for future clinical translation, which can be added in the field or on-site during sample collection and transferred or stored for processing, preserving the active fluorescence form for later reading. Despite the fast reactivity of the sensor, we chose to apply a 30 minute interval on ice before processing blood samples to simulate the clinical scenario whereby blood collected in a clinic room or at the hospital bedside needs to be transported to the laboratory before processing and analysis. Furthermore, we observed that after initial fluorescence readings were taken from Ru(bpy)_2_(dabpy)]^2+^ added samples, the addition of NOC13 still resulted in increased fluorescence counts (mean of 1.8 fold vs Ru(bpy)_2_(dabpy)]^2+^-only, *p* = *0.03*, paired *t-*test), showing that active sensor remained present in the plasma sample throughout the entire process of freeze-thawing (Fig. 8e). The significant 13.7% reduction in the fluorescent count (*p* = *0.005*, paired *t-*test) observed in the control experiments using the same processing conditions, incorporating a single freeze-thaw cycle with rabbit blood and [Ru(bpy)_2_(dabpy)]^2+^ with or without the NO scavenger cPTIO, suggested that the [Ru(bpy)_2_(T-bpy)]^2+^ complex can be used to specifically detect and estimate NO/NO adduct levels in plasma within a reasonable time-frame (Fig. 8f).

We observed evidence of some haemolysis (as relative changes in the sample colour) in two plasma samples that were used in the study. However, the haemolysis was independent of the incubation with [Ru(bpy)_2_(dabpy)]^2+^. The plasma samples with haemolysis reported relatively lower fluorescence counts initially and also after the addition of NOC13. This important observation of a relative reduction of fluorescence in the presence of haemolysis in rabbit blood/plasma suggests several underlying mechanisms. Extracellular free-iron, haem and haemoglobin are well-studied NO scavengers^39^ released during haemolysis, and may bind with NO to create haemoglobin-NO complexes and reduce the amount of NO availability for [Ru(bpy)_2_(dabpy)]^2+^. This phenomenon also may apply with the use of frozen and stored blood or plasma samples, similar to low NO availability being an underlying mechanism in old blood transfusion lesions^40^. Alternatively, haemoglobin being a photo-excitable molecule, could also interfere with the spectrophotometer reading of the bound NO-[Ru(bpy)_2_(T-bpy)]^2+^ complex. Therefore, corrections for haemolysis need to be addressed in future blood-based applications of [Ru(bpy)_2_(dabpy)]^2+^.

### Limitations

A few confounding factors and limitations for NO detection were identified during our assessments of [Ru(bpy)_2_(dabpy)]^2+^ as an *in vitro* and *ex vivo* NO sensor. In all fluorescence-based readings, we observed that the [Ru(bpy)_2_(dabpy)]^2+^ sensor itself caused a small baseline, concentration-dependent signal, which we corrected for by reporting normalised fluorescence counts throughout cell culture studies. These baseline contributions to the NO reading could be due to fluorescence of the inactive sensor itself as suggested by the difference in fluorescence counts between 10 µM and 50 µM [Ru(bpy)_2_(dabpy)]^2+^. It could also be due to reaction with gas phase NO, constitutive expression of NO in unstimulated endothelial cells or the sensor reactions with NO/metabolites in media. We observed considerable variability of baseline readings between independent experiments, but not within sample replicates or within different samples analysed in the same experiment. This is an inherent characteristic of plate reader-based experiments that require normalisation to the baseline and/or calibration using a standard curve in future applications of this sensor. Similarly, we also observed a continuing increase of fluorescence signal in culture media over time, as opposed to it stabilising rapidly in PBS. This suggests the need to limit the sensor-NO reaction to a specific time, prior to comparing readings between different biological samples, which we achieved for rabbit plasma by snap-freezing prior to reading.

### Future applications

We have demonstrated the application of a ruthenium-based molecular sensor, [Ru(bpy)_2_(dabpy)]^2+^ to detect NO by measuring the fluorescence of its active form [Ru(bpy)_2_(T-bpy)]^2+^ using spectrophotometry in cell-free media, in endothelial cell culture and in plasma. Some characteristics of [Ru(bpy)_2_(dabpy)]^2+^ that are relevant to its future application as an NO sensing tool in the clinic include its: (1) irreversible, cumulative NO detection capacity; (2) capacity to detect extracellular or supernatant NO in endothelial cells; (3) high baseline fluorescence which necessitates calculation of a change in fluorescence (∆fluorescence) as the comparative measurement across samples; and (4) time-dependent increase in fluorescence in biological samples that requires rate limiting steps. Within the limitations of the current study, [Ru(bpy)_2_(dabpy)]^2+^ is able to qualitatively differentiate the abundance of NO, with a limited ability to quantitatively differentiate µM concentrations. Future calibration experiments to develop concentration response curves to determine the NO sensitivity are needed to enhance the accuracy of these quantitative measurements. As the outcomes of the current study are limited by the sensitivity of spectrophotometry, alternative technologies for signal quantification such as luminescence, confocal microscopy and flow cytometry can be applied to further enhance the sensitivity and the application of this sensor. Interactions with plasma proteins, formation of protein corona around these [Ru(bpy)_2_(dabpy)]^2+^ molecules^41^ and the effects of such modifications on NO-sensor interface also need to be addressed in such validation studies. Ruthenium-based complexes are being developed for theranostic applications in cancer^42^, including Ruthenium-NO complexes which release NO^43^. Similar concepts could be applied intravascularly, to design NO-releasing Ruthenium-based complexes that are sensitive to NO availability inside blood vessels, particularly with atherosclerosis and cancer metastasis associated angiogenesis.

In conclusion, we were able to demonstrate significant and source-dependent (acetylcholine <H_2_O_2_≪NOC13) differences in ∆fluorescence with [Ru(bpy)_2_(T-bpy)]^2+^, suggesting the capability of this sensor to experimentally detect endogenous NO production by HUVECs, as well as exogenous NO present in cell supernatant. Magnitude of these changes in fluorescence under each condition were different but were comparable across fluorescence intensity changes with DAF-FM-diacetate, a commercially available direct NO sensor, and with the nitrite-based indirect NO measurements in the Griess assay. We also report a basic application of this sensor to detect NO in rabbit blood, suggesting its potential applicability as a candidate sensor for future probe or device development, to detect clinically measurable changes in NO. The sensitivity, sensor characteristics and concentration range of detection suggest potential future applications of the sensor for monitoring endogenous NO levels in various pathological conditions and also for monitoring bioavailability or compliance with pharmacological NO donors used in vascular diseases.

## Methods

### [Ru(bpy)_2_(dabpy)]^2+^

The ruthenium-based NO sensor complex bis(2,2′-bipyridine)(4-(3,4-diaminophenoxy)-2,2′-bipyridine)ruthenium(II)hexafluorophosphate ([(Ru(bpy)_2_(dabpy)][PF_6_]_2_) has been previously described^23^. Working solutions of 1 mM [Ru(bpy)_2_(dabpy)]^2+^ containing 0.1% dimethyl sulfoxide in phosphate buffered saline (PBS) were made and serial dilutions prepared as required. Either SynergyMx Microplate Reader (BioTek) or Glomax Discover System (Promega) were used for spectrophotometric readings and specified accordingly in each figure of the results.

### Detecting NO in cell-free media

Spectrophotometry was used to assess the comparative ability of 10 and 50 µM [Ru(bpy)_2_(dabpy)]^2+^ to detect NO under experimental conditions in cell-free PBS and phenol red-free M199 cell culture media with 1 mM NOC13 (1-Hydroxy-2-oxo-3-(3-amino-propyl)-3-methyl-1-triazene), an NO donor (T_1/2_ = 13.7 min, stoichiometry for NOC13:NO is 1:2) produced in our laboratory as previously described^44^. Based on this assessment, 50 µM [Ru(bpy)_2_(dabpy)]^2+^ was selected unless otherwise specified for further studies. The sensitivity of 50 µM [Ru(bpy)_2_(dabpy)]^2+^ to NO was assessed using spectrophotometry in cell-free PBS with NOC13 (10–40 µM), along with relevant sensor-free negative controls. Quadruplicate readings at λ_ex_ = 450 nm and λ_em_ = 590, 605, 615 and 630 nm were taken initially at 5 minutes with NOC13 and repeated after 2 hours. Subsequently, the response of [Ru(bpy)_2_(dabpy)]^2+^ to ascending concentrations of NO was assessed using 2–100 µM NOC5 (3-(Aminopropyl)-1-hydroxy-3-isopropyl-2-oxo-1-triazene, Sigma-Aldrich), an NO donor (T_1/2_ = 93 min), done in triplicate and readings taken over 93 minutes. Following these assessments, λ_ex_ = 450 nm and λ_em_ = 615 nm wavelengths were used unless otherwise specified for all spectrophotometric readings with cell-free media, cells or plasma.

In a separate experiment, the concentration of cPTIO (2-(4-Carboxyphenyl)-4,4,5,5-tetramethylimidazoline-1-oxyl-3-oxide, Sigma-Aldrich), a NO scavenger, was increased from 5–200 µM in phenol red-free cell culture media with 10 µM [Ru(bpy)_2_(dabpy)]^2+^. The plate was read 30 minutes after addition of NOC13 (1 mM). All readings were normalised to the initial reading at each concentration of cPTIO to account for background contributions from cPTIO. These readings were repeated in PBS in the presence and absence of a 200 µM cPTIO and 10 µM [Ru(bpy)_2_(dabpy)]^2+^ before and up to 20 minutes after addition of excess NOC13.

Nitric oxide sensitivity of [Ru(bpy)_2_(dabpy)]^2+^ was compared with a conventional, direct NO indicator, DAF-FM-diacetate (4-Amino-5-Methylamino-2′, 7′-Difluorofluorescein Diacetate, Life Technologies, Australia). Quadruplicate readings were taken in PBS with 0.01–10 µM NOC13 (15 min) and 10 µM [Ru(bpy)_2_(dabpy)]^2+^ (λ_ex_ = 450 nm and λ_em_ = 615 nm) or 10 µM DAF-FM-diacetate (λ_ex_ = 495 nm and λ_em_ = 515 nm). Griess assay (Nitrite/Nitrate Assay Kit, Sigma-Aldrich) was used according to the manufacturer’s instructions to determine NO levels indirectly and compare the sensitivity of [Ru(bpy)_2_(dabpy)]^2+^. The specific amounts of NOC13 was calculated to release 0.1–10 µM NO in PBS, within 2 hours of the assay. Absorbance at 560 nm was measured Glomax Discover System.

### Human Umbilical Vein Endothelial Cells (HUVECs)

HUVECs were isolated from umbilical cords obtained from consenting donors, as approved by the Human Research Ethics Committee of the Royal Adelaide Hospital, Adelaide, South Australia. Informed, written consent was obtained from subjects in accordance with the ‘Declaration of Helsinki’ and all procedures were performed in accordance with the National Statement on Ethical Conduct in Human Research (2007) in Australia. Primary HUVECs were extracted by collagenase digestion at 37 °C and isolated in RPMI (Roswell Park Memorial Institute) media (Gibco, ThermoFisher-Scientific) as previously described^45,46^. HUVECs were cultured using M199 growth media with 20% foetal bovine serum (FBS) or MesoEndo Cell Growth Medium (Sigma-Aldrich) in a cell incubator at 37 °C with humidified 5% carbon dioxide, and used for assays when >90% confluent. Between the passages and for assays with suspended cells, 0.25% trypsin was used for cell detachment, neutralized with cell culture media containing 20% FBS, centrifuged at 4 °C, 220rcf for 5 minutes and washed with sterile PBS. Primary HUVECs from passages 2–6 were used to detect NO in endothelial cells.

### Endothelial Cell viability and function in the presence of [Ru(bpy)_2_(dabpy)]^2+^

Water Soluble Tetrazolium-1 (WST-1) reagent (Roche, NSW, Australia) based colorimetric assay was used to assess the effect of up to 72 hours of exposure to [Ru(bpy)_2_(dabpy)]^2+^ on cell viability before using it for NO sensing in HUVECs. Four time points of exposure were used for the viability assessment; (1)2 h with [Ru(bpy)_2_(dabpy)]^2+^, washed with culture media and WST-1 assay after 24 h, (2)2 h with [Ru(bpy)_2_(dabpy)]^2+^, washed with culture media and WST-1 assay after 72 h (3)24 h with [Ru(bpy)_2_(dabpy)]^2+^, WST-1 assay at the end of exposure, and (4)72 h with [Ru(bpy)_2_(dabpy)]^2+^, WST-1 assay at the end of exposure. HUVECs were then grown on Matrigel following 24 hour exposure to 10 and 50 µM [Ru(bpy)_2_(dabpy)]^2+^ to assess any effects on their angiogenic capacity. In addition, H_2_DCFDA (2′, 7′-dichlorodihydrofluorescein diacetate) was used to demonstrate any changes in general oxidative stress in response to [Ru(bpy)_2_(dabpy)]^2+^ (experimental details in Supplementary Section 1).

### Localisation of the NO sensor in HUVECs

Spectrophotometric and confocal microscopic analysis of HUVECs in the presence of [Ru(bpy)_2_(dabpy)]^2+^ and NOC13 before and after media changes was used to determine the intracellular vs extracellular localisation of active NO-bound [Ru(bpy)_2_(T-bpy)]^2+^ fluorescence. ICP-MS analysis was performed on cell and supernatant components with relevant controls to assess the uptake of Ruthenium by HUVECs (experimental details in Supplementary Section 2).

### Detection of endogenous NO using [Ru(bpy)_2_(dabpy)]^2+^ in HUVECs

Acetylcholine^13,47^ and hydrogen peroxide (H_2_O_2_)^36^ are well studied stimuli to induce phosphorylation of eNOS and increase endogenous NO production in endothelial cells. We confirmed these mechanisms in HUVECs under our experimental conditions, using Western Blot protein assessments of phosphorylated eNOS (pS1177), with increasing concentrations of these two agonists (experimental details in Supplementary Section 3). HUVECs were incubated with [Ru(bpy)_2_(dabpy)]^2+^ for 24 hours and subjected to spectrophotometry with increasing concentrations of acetylcholine (5–100 µM) or H_2_O_2_ (50–250 µM) in separate experiments. These experiments were repeated with 10 µM acetylcholine^13^ (15 min) or 150 µM H_2_O_2_^13^ (20 min) in HUVECs from different donors to account for biological variability. DAF-FM-diacetate (2 µM, 30 min) was used in parallel experiments as comparators to the direct NO detection capacity of [Ru(bpy)_2_(dabpy)]^2+^. Unwashed HUVECs were read with 10 µM acetylcholine (15 min) or 200 µM H_2_O_2_ (20 min)^13^, with and without 500 µM L-NAME^36^ (30 min), to demonstrate the reduction of fluorescence in the presence of an eNOS inhibitor.

### Detection of exogenous NO using [Ru(bpy)_2_(dabpy)]^2+^ in HUVECs

HUVECs were incubated with [Ru(bpy)_2_(dabpy)]^2+^ for 24 hours, subjected to spectrophotometry in the presence of an exogenous NO donor NOC13 (1 mM, 30 min) and repeated in HUVECs from different donors to account for biological variability. A direct, conventional NO assay using DAF-FM-diacetate (2 µM, 30 min) was used in parallel experiments as comparators to the direct exogenous NO detection capacity of [Ru(bpy)_2_(dabpy)]^2+^ in HUVECs using the same spectrophotometer.

### Detecting NO in plasma

All animal care and handling procedures were approved by Animal Ethics Committee of the South Australian Health and Medical Research Institute (SAHMRI) and the Animal Welfare Committee of the Flinders University, South Australia. All procedures were performed in accordance with the Australian Code for the Care and Use of Animals for Scientific Purposes (2013). New Zealand White, male rabbits were bred in-house at the SAHMRI Preclinical Imaging and Research Laboratory animal facility (Gilles Plains, South Australia) or in the Animal Facility of the Flinders University. Nine rabbits were anaesthetised at two years of age with intramuscular ketamine (35 mg/kg)/xylazine (5 mg/kg) and 2–5% isoflurane inhalation. After thoracotomy, blood was directly drawn from the heart in to a 1 mL syringe containing EDTA/PBS solution and either 500 µL of 100 µM [Ru(bpy)_2_(dabpy)]^2+^ in PBS or 500 µL PBS as the vehicle control without [Ru(bpy)_2_(dabpy)]^2+^. The samples were processed in two groups and all reading were done on the SynergyMx Microplate Reader at λ_ex_ = 450 nm and λ_em_ = 615 nm. Blood from four additional rabbits were used for control experiments with/without cPTIO, a scavenger for NO. Details of sample processing and analysis in each group along with relevant schemes are provided in Supplementary Section 4.

#### Statistical analysis

Data were analysed using GraphPad Prism software (GraphPad Software, Inc. La Jolla, CA, USA). Shapiro-Wilk normality test was used to test the normality of data distribution. Paired or unpaired *t-test* was used where relevant to compare the fluorescence counts under different conditions and the two-tailed *p-value* for significance was <0.05. One-way ANOVA was used to compare three or more groups and the *p-values* for significant differences were derived from a relevant post-hoc test for multiple comparisons. All data are reported as mean ± standard deviation (s.d.), along with the individual data points where relevant to demonstrate the biological variability.

## Supplementary information

Supplementary Information

## Data Availability

The datasets generated or analysed on the current study are available on reasonable request from the corresponding author.
