# Supplementary material for: A Novel Ruthenium-based Molecular Sensor to Detect Endothelial Nitric Oxide
Source: Sci Rep. 2019 Feb 8;9:1720. doi: 10.1038/s41598-019-39123-3 (PMC6368587; doi:10.1038/s41598-019-39123-3)
Supplement: Supplementary file 1 — Supplementary Information [file 41598_2019_39123_MOESM1_ESM.pdf]

Supplementary data section for

A Novel Ruthenium-based Molecular Sensor to Detect Endothelial Nitric Oxide

Authors:

Achini K Vidanapathirana [1, 2, 4, 5 †]
Benjamin J Pullen [1, 2, 4, 5 †]
Run Zhang [2, 3]
MyNgan Duong [1, 2, 4]
Jarrad M. Goyne [1, 2, 4, 5]
Xiaozhou Zhang [2, 5, 6]
Claudine S Bonder [4, 7]
Andrew D. Abell [2, 5, 6]
Christina A Bursill [1, 2, 4]
Stephen J Nicholls [1, 2, 4, ‡]
Peter J Psaltis [1, 2, 4*‡]

[†]Equal contributions
[‡]Joint senior authors

* Address for Correspondence
Peter J Psaltis, MBBS PhD
Vascular Research Centre, Heart Health Theme, Level 6
South Australian Health and Medical Research Institute, North Terrace, Adelaide, South Australia, Australia, 5000
peter.psaltis@sahmri.com

Affiliations

[1] Vascular Research Centre, Heart Health Theme, South Australian Health and Medical Research Institute (SAHMRI), Adelaide, South Australia 5000, Australia
[2] Australian Research Council (ARC) Centre of Excellence for Nanoscale BioPhotonics (CNBP), Adelaide, Australia
[3] Australian Institute for Bioengineering and Nanotechnology (AIBN), University of Queensland, St Lucia, Queensland 4072, Australia
[4] Adelaide Medical School, University of Adelaide, Adelaide, South Australia 5000, Australia.
[5]Institute for Photonics and Advanced Sensing (IPAS), School of Physical Sciences. University of Adelaide, Adelaide, South Australia 5000, Australia.
[6] Department of Chemistry, University of Adelaide, Adelaide, South Australia 5000, Australia.
[7] Centre for Cancer Biology, SA Pathology and University of South Australia, Adelaide, South Australia 5000, Australia.

Supplementary Figure S1

Chemical structures and reactivity of [Ru(bpy)$_2$(dabpy)]$^{2+}$ and [Ru(bpy)2(T-bpy)]$^{2+}$ used as the nitric oxide sensor in the study

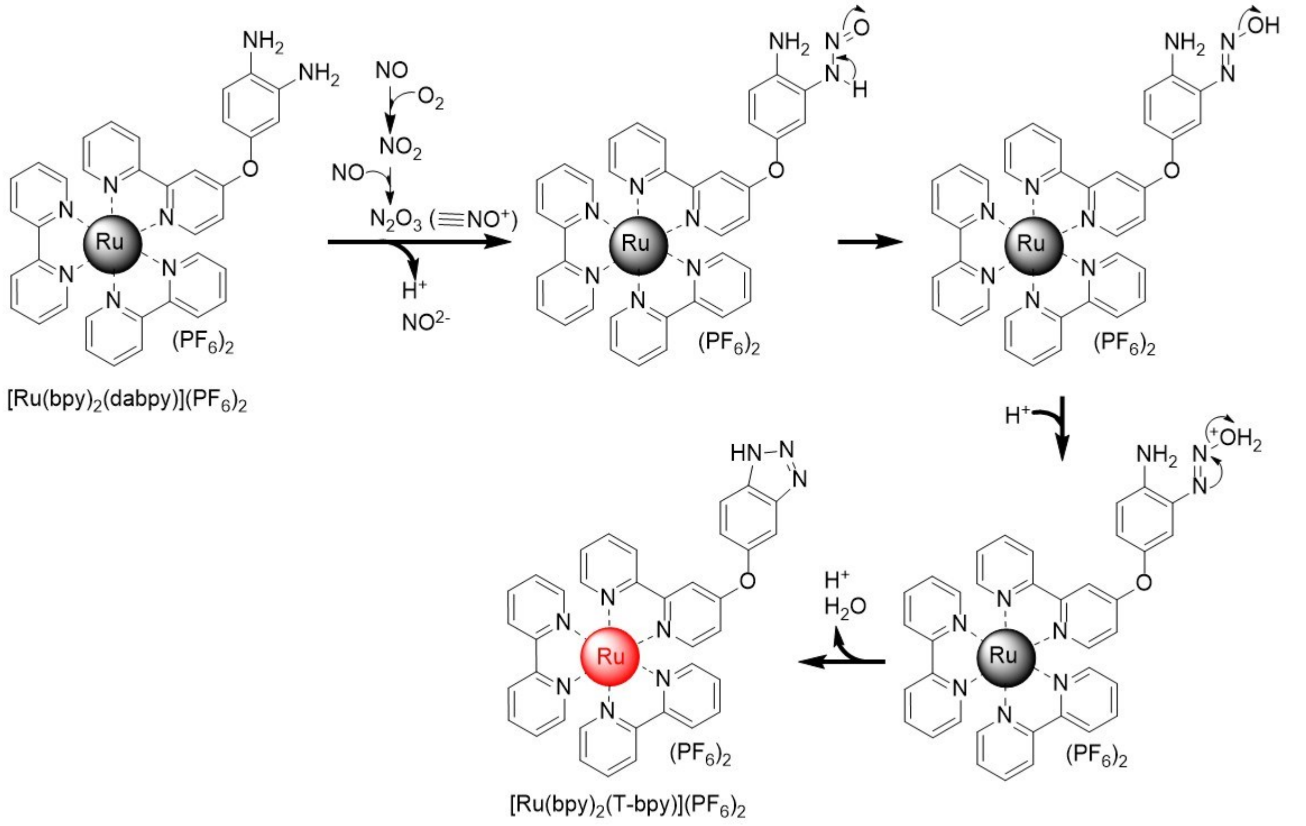

Supplementary Figure S2

Comparison of fluorescence counts from two batches of $Ru(bpy)_2(dabpy)]^{2+}$ working solutions in PBS and cell culture media.

(**a, b**) Baseline fluorescence counts under λ_{ex}=450 nm and λ_{em}=615 nm on the Glomax Discover System from two working solutions (identified as A and B, where A was refrigerated and stored after reconstituting) of 10 µM and 50 µM $[Ru(bpy)_2(dabpy)]^{2+}$ in cell-free PBS and phenol red-free M199 cell culture media. The *p-values* were derived from one-way ANOVA followed by Tukey's multiple comparisons test and only the *$p<0.05$* values are reported. (**c, d**) Representative fluorescence count readings over 60 minutes under λ_{ex}=450 nm and λ_{em}=615 nm, after the addition of NOC13 (1 mM) to 10 µM or 50 µM $[Ru(bpy)_2(dabpy)]^{2+}$ from the two working solutions in cell-free PBS and in phenol red-free M199 cell culture media. All data are represented as mean ± s.d. from 3-6 cell-free replicates.

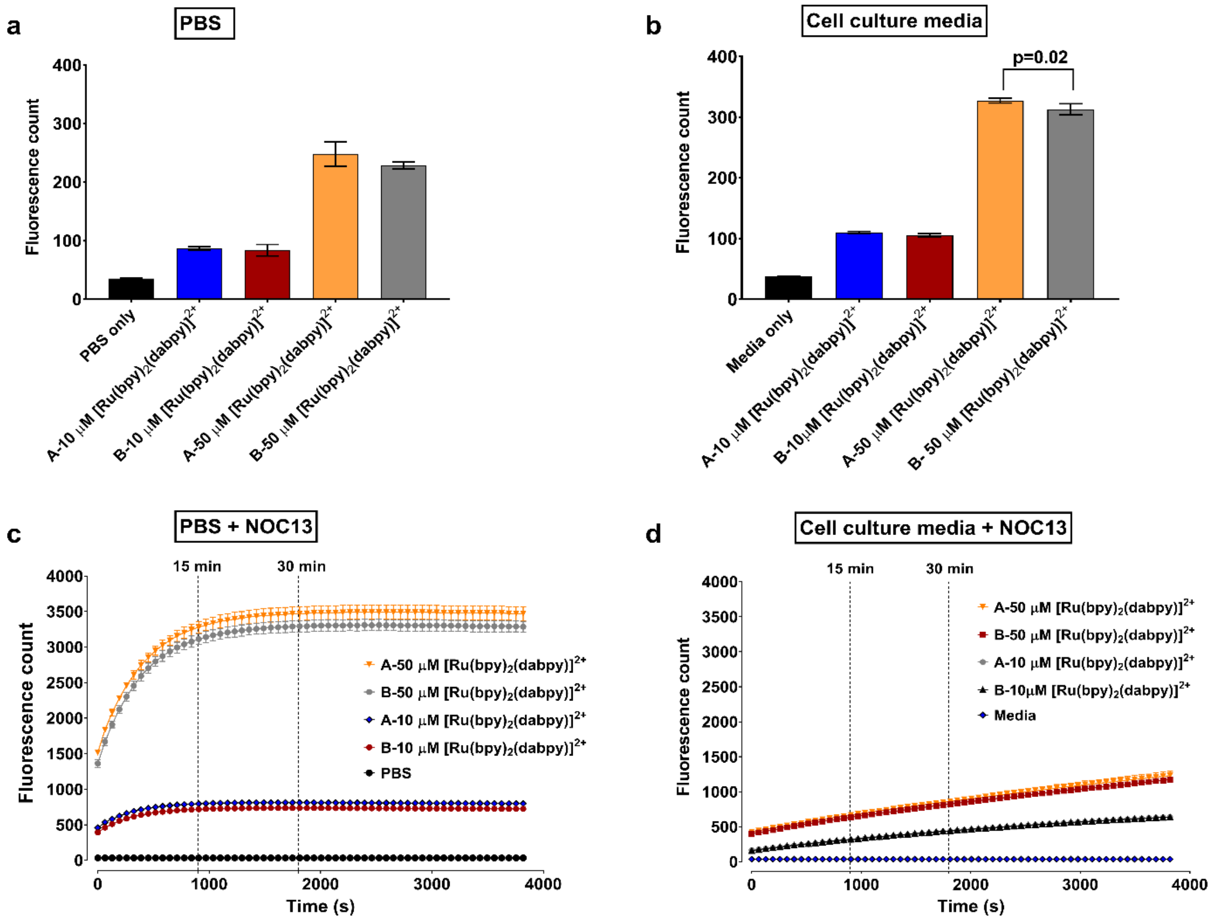

Supplementary Figure S3

Concentration dependent changes in fluorescence counts with $[Ru(bpy)_2(dabpy)]^{2+}$ over time with an NO donor, NOC5.

(**a**) Fluorescence counts under λ_{ex}=450 nm and λ_{em}=615 nm on the Glomax Discover System with 2-100 μM NOC5 and 10 μM $[Ru(bpy)_2(dabpy)]^{2+}$ in PBS with readings recorded over 90 minutes. (**b**) Approximate concentration of NO in the PBS solution after 30 and 90 minutes of addition of NOC5, calculated based on the half-life of 93 minutes at a room temperature of 22˚C. Concentration dependent changes in the fluorescence at (**c**) 30 minutes and (**d**) 90 minutes after the addition of NOC5. The discontinuous lines represent the best fit used for the regression analysis and to calculate the coefficient of determination (R^2) for each concentration dependent response. All data are represented as mean ± s.d. from 3 cell-free replicates.

a

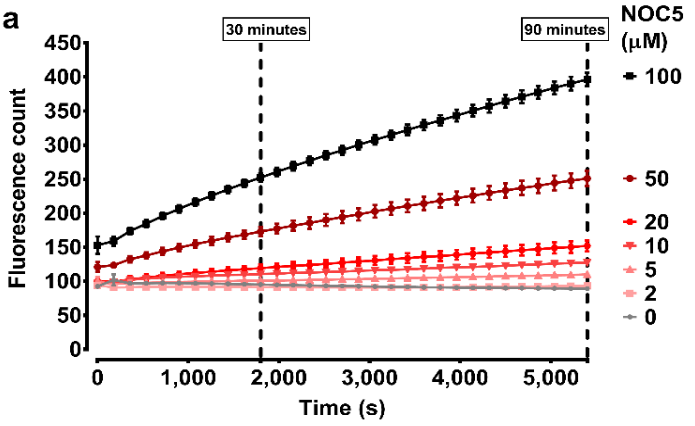

b

[NOC5]μM	[NO]μM at 30 min	[NO]μM at 90 min
0	0.000	0.000
2	0.801	1.955
5	2.004	4.887
10	4.007	9.774
20	8.014	19.548
50	20.036	48.869
100	40.072	97.738

c d

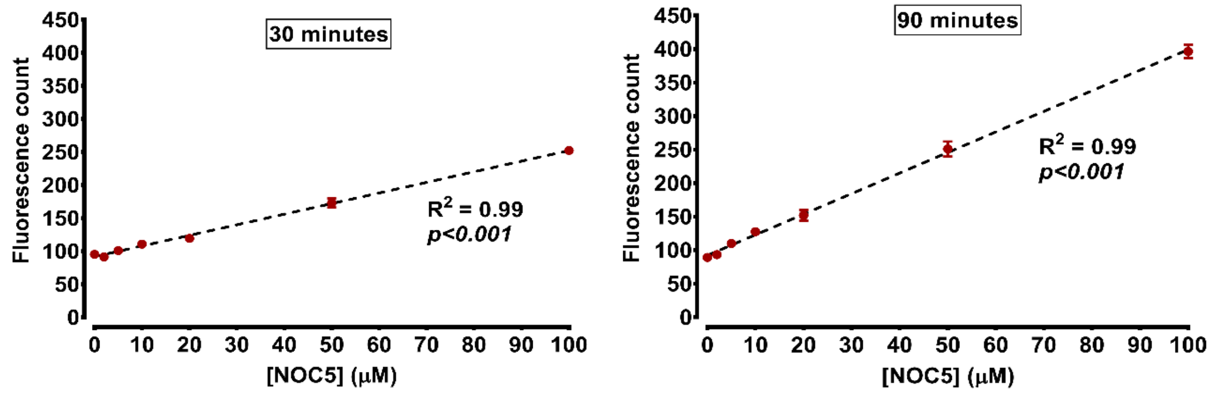

Supplementary Figure S4

Background controls with acetylcholine.

(**a**, **b**) Fluorescence count readings under λ_{ex}=450 nm and λ_{em}=615 nm on the Glomax Discover System 30 minutes after the addition of 10 µM acetylcholine (Ach) to cell-free PBS or culture media with 10 µM and 50 µM $[Ru(bpy)_2(dabpy)]^{2+}$. The *p-values* were derived from one-way ANOVA followed by Tukey's multiple comparisons test and the differences in the fluorescence count after the addition of Ach were not statistically significant. (**c**, **d**) Fluorescence count readings after the addition of NOC 13 to different sensor concentrations with and without 10 µM Ach in PBS or cell culture media, followed up for 60 minutes. All data are represented as mean ± s.d. from 3 cell-free replicates.

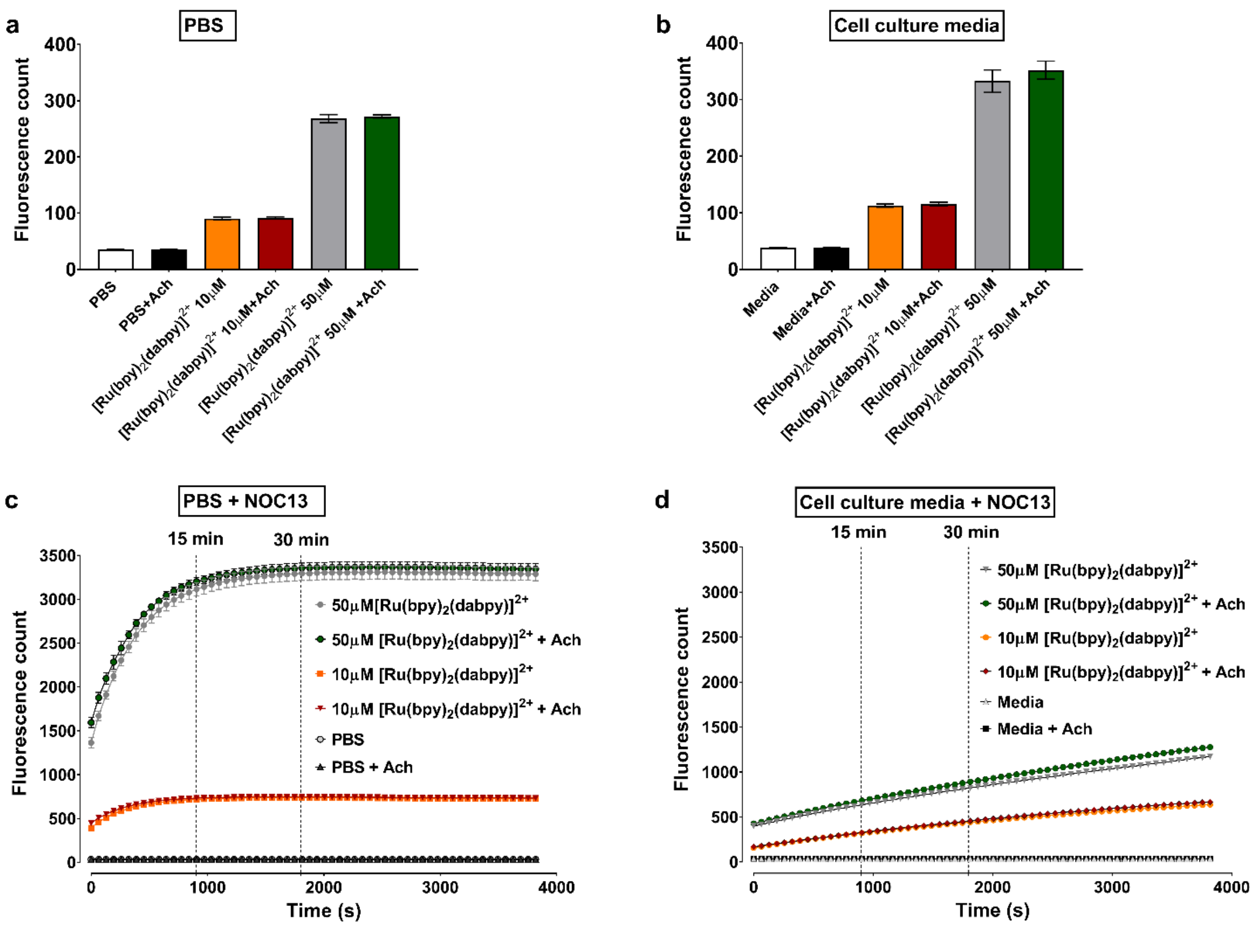

Supplementary Figure S5

Background controls with hydrogen peroxide.

(**a, b**) Fluorescence count readings under λ_{ex}=450 nm and λ_{em}=615 nm on the Glomax Discover System 30 minutes after the addition of 150 µM hydrogen peroxide (H_2O_2) to cell-free PBS or culture media with 10 µM and 50 µM $[Ru(bpy)_2(dabpy)]^{2+}$. The *p-values* were derived from one-way ANOVA followed by Tukey's multiple comparisons test and only the *p<0.05* values are reported. (**c, d**) Fluorescence count readings after the addition of NOC 13 to different sensor concentrations with and without 150 µM H_2O_2 in (**c**) PBS or (**d**) cell culture media followed up for 60 minutes. All data are represented as mean ± s.d. from 3 cell-free replicates.

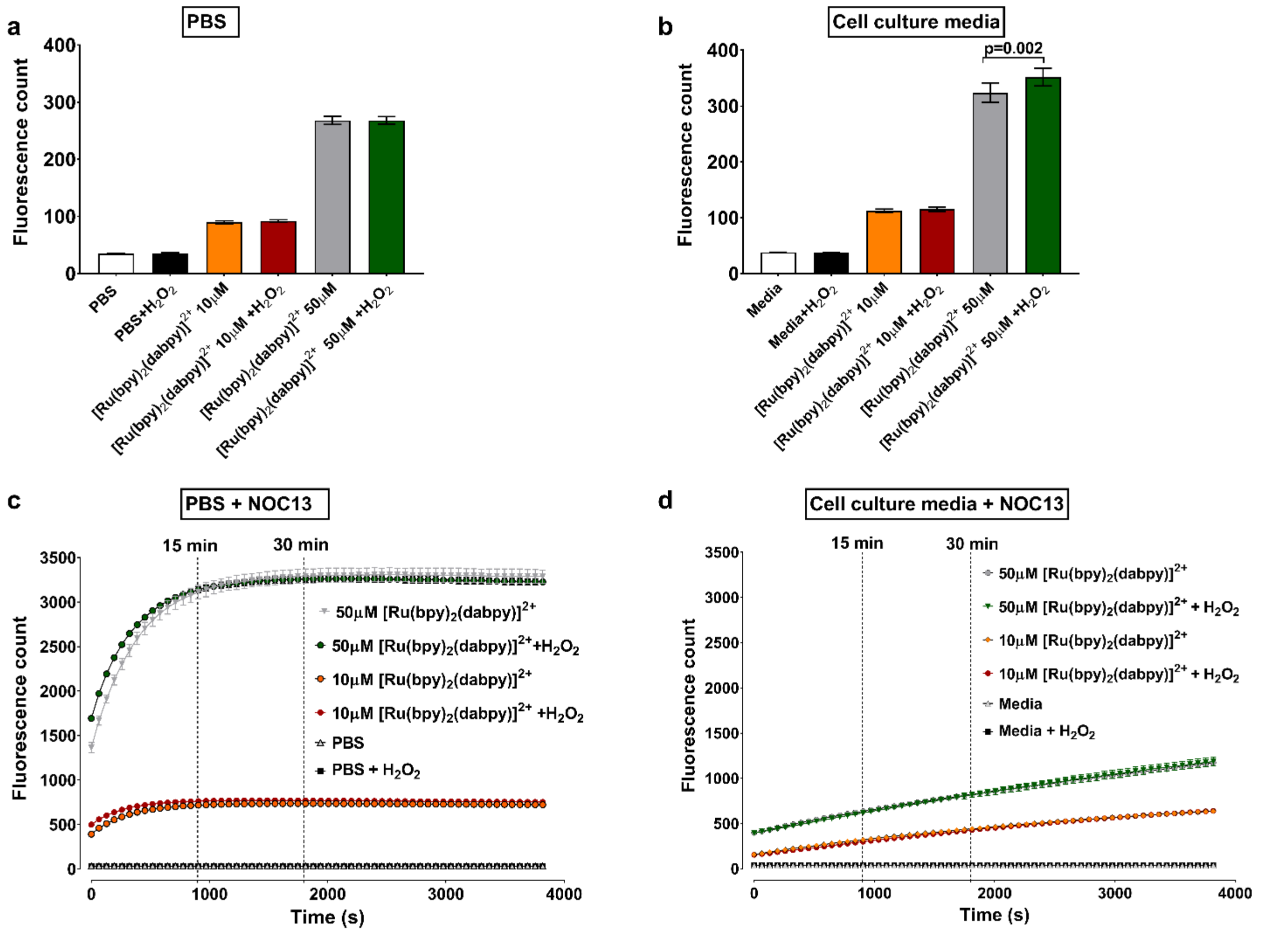

Supplementary Figure S6

Background controls to assess the interactions between hydrogen peroxide and $[Ru(bpy)_2(dabpy)]^{2+}$

Fluorescent count readings (mean ± s.d.) under λ_{ex}=450 nm and λ_{em}=615 nm on the Glomax Discover System 5 minutes after the addition of 0-500 µM hydrogen peroxide (H_2O_2) in PBS or phenol red-free M199 cell culture media in the presence of 10 µM $[Ru(bpy)_2(dabpy)]^{2+}$.

In the last condition, 200 µM of cPTIO was added to 150 µM H_2O_2, to demonstrate the reduction of the fluorescent count in the presence of a nitric oxide scavenger, despite the presence of H_2O_2.

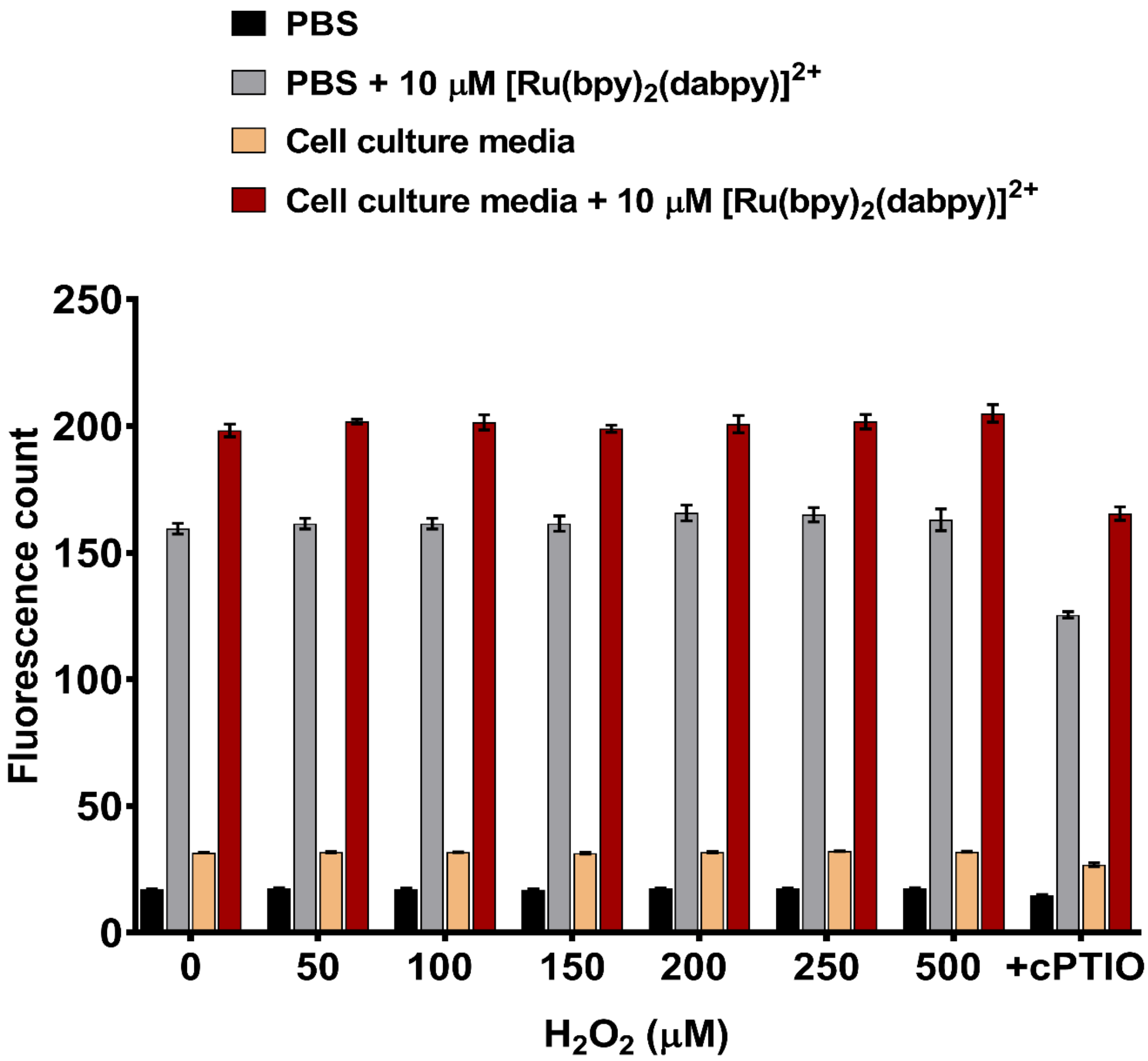

Supplementary Figure S7

Indirect detection of NO using the Griess assay in HUVECs.

Representative results (mean ± s.d. of replicate readings) demonstrating the absorbance readings in the Griess assay with 10 µM acetylcholine (Ach, 15 min) and 150 µM hydrogen peroxide (H_2O_2, 150 min) as endogenous stimuli and with NOC13 as a source of exogenous NO in HUVECs at 37˚C and 5% CO_2. The absorbance readings for (**a**) nitrites only and (**b**) total nitrites+nitrates are reported. The absorbance at 40 µM NO was calculated using the standard curves derived using the standard solutions provided in the kit by the manufacturer.

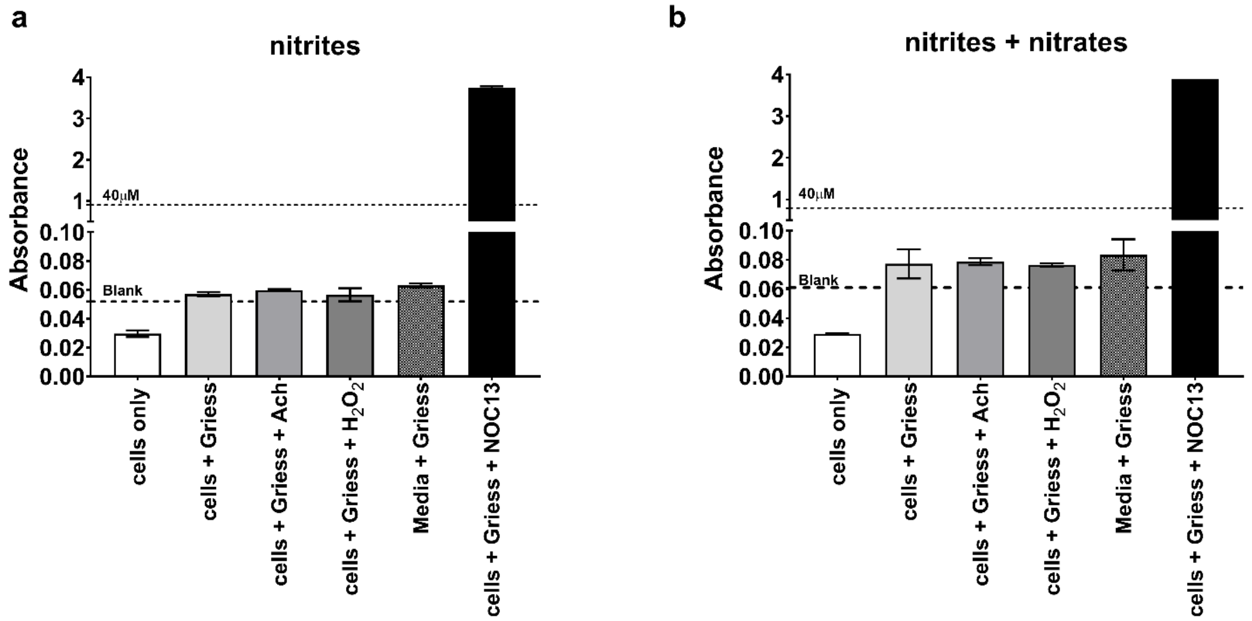

Supplementary Information - Section 1

Assessing the effects of [Ru(bpy)$_2$(dabpy)]$^{2+}$ exposure on endothelial cell viability, function and proliferation

WST-1 assay

Water Soluble Tetrazolium 1 (WST-1) reagent (Roche, NSW, Australia) based colorimetric assay was used to assess the effect of up to 72 hours of exposure to [Ru(bpy)$_2$(dabpy)]$^{2+}$ on cell viability before using it for NO sensing in HUVECs. The optimum cell density and incubation period with WST-1 was determined by preliminary experiments. HUVECs in passage 3 were seeded at 4000 cells/well using Fluorescence Activated Cell Sorting into 96 well plates pre-coated with gelatine. HUVECs were incubated overnight in a cell incubator at 37˚C and 5% CO_2 with cell culture media and 0.001-200 µM [Ru(bpy)$_2$(dabpy)]$^{2+}$ was added. All exposures were done in quadruplicate and cell-free, gelatine, and sensor only controls were matched for all concentrations. Cell culture media was replaced with 100 µl of phenol red-free RPMI cell culture media and 10 µl of WST-1 reagent was added to each well at the end of each time point. HUVECs were incubated for 4 hours at 37˚C and 5% CO_2 and read using an iMark microplate reader (BIORAD) at 440 nm.

Four time points of exposure were used for the viability assessment:

1). 2 h with [Ru(bpy)$_2$(dabpy)]$^{2+}$, washed with culture media and WST-1 assay after 24 h
2). 2 h with [Ru(bpy)$_2$(dabpy)]$^{2+}$, washed with culture media and WST-1 assay after 72 h
3). 24 h with [Ru(bpy)$_2$(dabpy)]$^{2+}$, WST-1 assay at the end of exposure
4). 72 h with [Ru(bpy)$_2$(dabpy)]$^{2+}$, WST-1 assay at the end of exposure

Assessment of angiogenic (vascular tube formation) capacity of the HUVECs following exposure $[Ru(bpy)_2(dabpy)]^{2+}$

HUVECs in passage 2 were seeded in six well plates ($1.2x10^5$ cells/well) and were used for the experiments when 90% confluent. HUVECs were incubated with either PBS, 10 or 50 µM $[Ru(bpy)_2(dabpy)]^{2+}$ for 24 hours in Meso-endo media at 37 °C, 5% CO_2. The Meso-Endo media was removed from the plated cells and washed briefly with filtered 1X PBS. The cells were trypsinised with 700 µL trypsin-EDTA to each well, incubated for 5 min at 37°C followed by inactivation with equal volume of Meso-Endo media. The cells were centrifuged at 2500 rpm for 5 min, re-suspended in 1 mL of Meso-Endo media and counted for each condition. A cell suspension of 1.2×10^5 cells/mL with total volume 2.6 mL in Meso-Endo media was made for each condition. Matrigel (Corning Matrigel Growth Factor Reduced (GFR) Basement Membrane Matrix) was added to each experimental well (40 µL) in quadruplicate in a 96-well plate under sterile conditions and kept on ice. The suspension of cells of 200 µL (1.2×10^4cells/well) was added per well in Meso-Endo. The plates were incubated at 37°C, 5% CO_2 6 and 24 hours and imaged using an AX10 Zeiss microscope. Two images (central and peripheral) were taken from each quadrant in a well. Counting of the number of branches and branch points was performed using Image J software (National Institutes of Health, USA).

__Supplementary Figure S8__

Representative images from the Matrigel assay

Representative images of the cell culture wells of the Matrigel assay under normoxic conditions following 24 hours pre-exposure to $[Ru(bpy)_2(dabpy)]^{2+}$
Magnification **X 2.5**

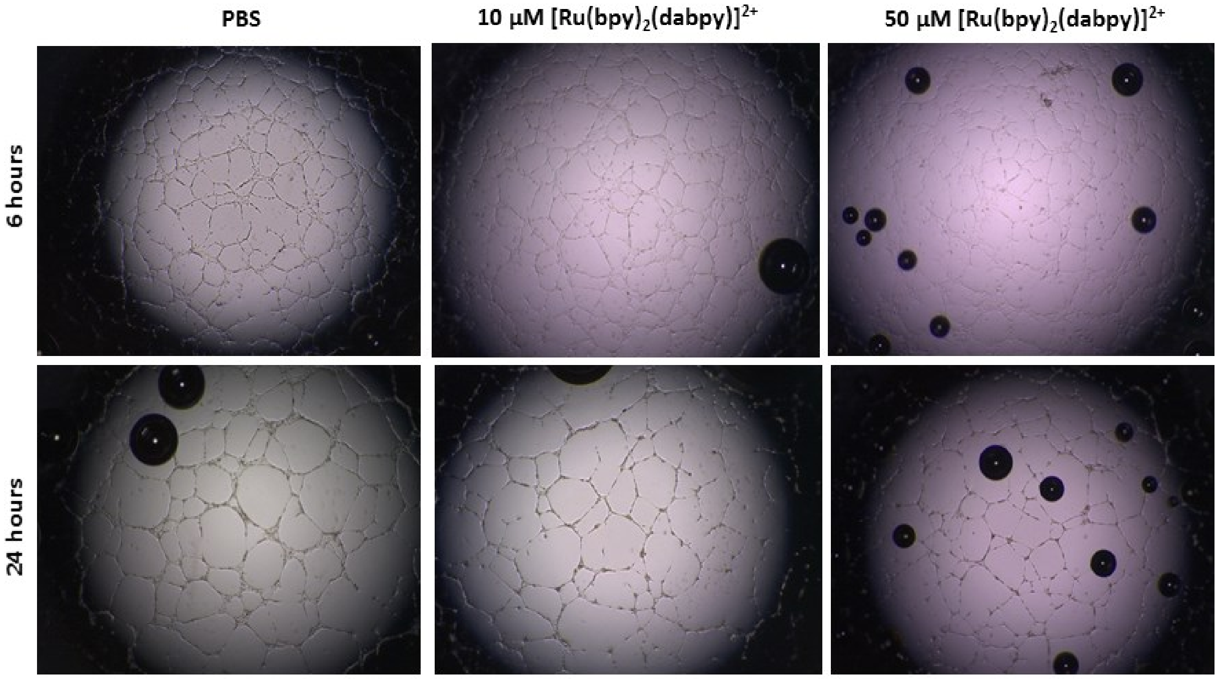

Assessment of changes in general oxidative stress in response to [Ru(bpy)$_2$(dabpy)]$^{2+}$

H$_2$DCFDA (2',7'-dichlorodihydrofluorescein diacetate, Thermo Fisher Scientific Australia) was used to assess the changes in the abundance of reactive oxygen species with the exposure to [Ru(bpy)$_2$(dabpy)]$^{2+}$. HUVECs were plated on a 96 well plate (5000 cells/well) and left overnight. Cells were loaded for 10 min with 5 µM H$_2$DCFDA in phenol-red-free medium in the dark and then treated with 10 or 50 µM [Ru(bpy)$_2$(dabpy)]$^{2+}$. A 5 µM H$_2$DCFDA only condition was used as the baseline control, along with 500 µM H_2O_2 treated HUVECs with 5 µM H$_2$DCFDA as the positive control. The cells were incubated at 37 °C, 5% CO_2 and the fluorescent count readings from the by-product DCF (2',7'-dichlorofluorescein) were taken 5, 10, 15, 30 and 60 minutes under λ_{ex}=475 nm and λ_{em}=500-550 nm on the Glomax Discover System.

__Supplementary Figure S9__

Changes in general oxidative stress in response to [Ru(bpy)$_2$(dabpy)]$^{2+}$

Fluorescent count readings (mean ± s.d.) under λ_{ex}=475 nm and λ_{em}=500-550 nm on the Glomax Discover System 5, 10, 15, 30 and 60 minutes after incubation at 37 °C, 5% CO_2 with H$_2$DCFDA and 10 or 50 µM [Ru(bpy)$_2$(dabpy)]$^{2+}$. 500 µM H_2O_2 treated HUVECs with 5 µM H$_2$DCFDA was used as the positive control for oxidative stress. Changes in the by-product DCF fluorescence overtime in **(a)** cell free media with and **(b)** HUVECs with [Ru(bpy)$_2$(dabpy)]$^{2+}$. The *p-values* were derived from repeated measures one-way ANOVA followed by Tukey's multiple comparisons test and *denotes *p<0.05* compared to the H$_2$DCFDA only control at each time point.

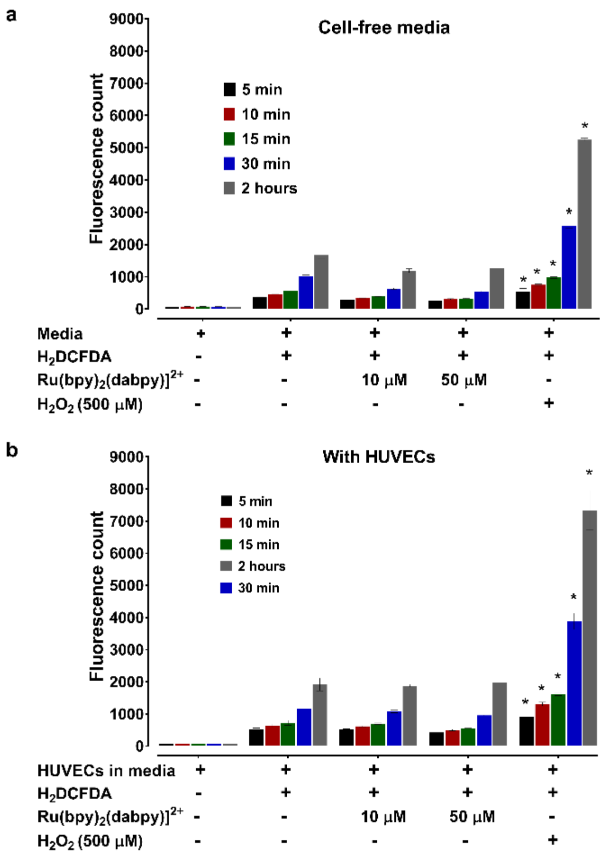

Supplementary Information - Section 2

Localisation of $[Ru(bpy)_2(dabpy)]^{2+}$ in HUVECs

Spectrophotometry

Spectrophotometry (SynergyMx Microplate Reader, BioTek) was used to analyse fluorescence signal in the supernatant of washed and unwashed HUVECs in passage 3 that were treated with 1 mM NOC13 or 10 µM acetylcholine (15 min) and 50 µM $[Ru(bpy)_2(dabpy)]^{2+}$.
The readings were taken in two groups:

1) cell culture media changed before reading the plate
2) no cell culture media change before the initial reading and subjected to two media changes with a reading after each change

All readings were in quadruplicate and repeated with $[Ru(bpy)_2(dabpy)]^{2+}$ incubation at 37 °C, 5% CO_2 for 4, 6 and 24 hours, read at λ_{ex}=450 nm and λ_{em}=590, 605, 615 and 630 nm. In addition, the supernatant was isolated during the first media change and the fluorescence count was measured to determine the location of the fluorescence signal as cell surface or supernatant based.

Supplementary Figure S10

Localisation of fluorescence from $[Ru(bpy)_2(T\text{-}bpy)]^{2+}$ in HUVECs using spectrophotometry.

Fluorescence counts (mean ± s.d.) on the SynergyMx Microplate Reader from a representative quadruplicate experiment at λ_{ex}=450 nm and λ_{em}=615 nm for HUVECs under 3 conditions, 24 hours after incubation with 50 µM $[Ru(bpy)_2(dabpy)]^{2+}$ and 10 µM acetylcholine (Ach) or excess NOC13.

1. **No media change** – HUVECs undisturbed and read after the addition of $[Ru(bpy)_2(dabpy)]^{2+}$, Ach or NOC13.
2. **Media changed** – HUVECs which had the supernatant removed and media changed after the addition of $[Ru(bpy)_2(dabpy)]^{2+}$, Ach or NOC13.
3. **Supernatant** – The cell supernatant removed in condition (2) read in the same plate reader.

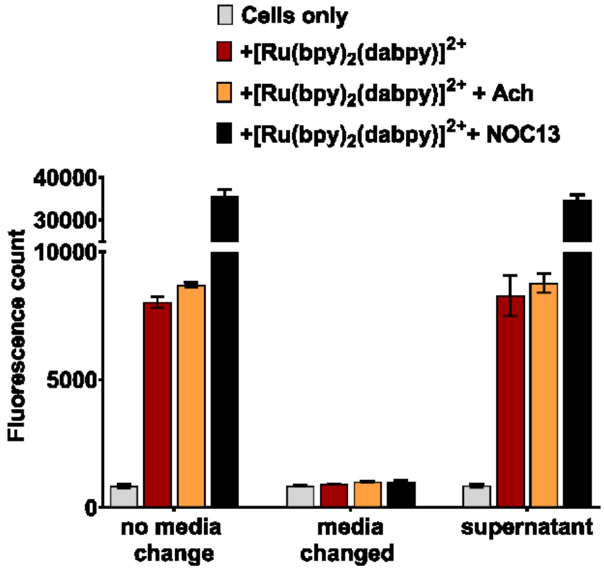

Confocal microscopy

HUVECs (20000 cells/well) were grown on gelatine coated 12 well glass bottom plates (Cell E&G, San Diego, CA, USA) and were incubated with 50 µM $[Ru(bpy)_2(dabpy)]^{2+}$ for 24 hours and treated with excess (1 mM) NOC13 (1-Hydroxy-2-oxo-3-(3-amino-propyl)-3-methyl-1-3\triazene, $T_{1/2}$ = 13.7 min) for 1 hour at 37˚C in an incubator or live imaging chamber attached to microscope set-up. A nucleic acid stain, 2 µM Hoechst 33342 (ThermoFisher) was added for 30 minutes before imaging to demarcate the nucleus. A Leica TCS SP8X/MP Confocal Microscope under λ_{ex}=473 nm and λ_{em}=565-645 nm was used for imaging, with analysis performed using LAS-X imaging software (Leica Microsystems Pty Ltd, NSW, Australia). Cells were imaged with and without changing the media to confirm the cellular localisation of the fluorescence. Fluorescence intensity of different channels were quantified in each image, from three different sections for each condition before and after media change.

In the second experiment, the HUVECs were incubated with 50 µM $[Ru(bpy)_2(dabpy)]^{2+}$ for 24 hours followed by 50 µM or 100 µM NOC13 for 1 hour at 37˚C in an incubator or live imaging chamber attached to microscope set-up. A nucleic acid stain, 2 µM Hoechst 33342 was added for 30 minutes before imaging. Cells were imaged with and without changing the media to confirm the cellular localisation of the fluorescence. Fluorescence intensity of different channels were quantified in each image, from three different sections for each condition before and after media change.

Mean intensity from the red channel ($[Ru(bpy)_2(dabpy)]^{2+}$) was normalized to the mean intensity of the blue channel (Hoechst), to determine the mean fluorescence relative to the number of cells.

Supplementary Figure S11

Localisation of fluorescence from NO bound $[Ru(bpy)_2(T\text{-}bpy)]^{2+}$ in HUVECs using confocal microscopy

$[Ru(bpy)_2(T\text{-}bpy)]^{2+}$ emits an extracellular signal with HUVECs as represented in the merged confocal images of HUVECs stained with Hoechst 33342 (a nuclear stain - Blue) and $[Ru(bpy)_2(T\text{-}bpy)]^{2+}$ (NO bound sensor – Red). HUVECs in the presence of NOC13 (an exogenous NO donor) with (**a-c**) no media change or (**d-f**) with a media change prior to imaging. Mean intensities from the red channels in three stacked images are reported (■) alongside the normalised intensities to the nuclear stain (■) in cells subjected to (**g**) no media change and (**h**) with the media changed.

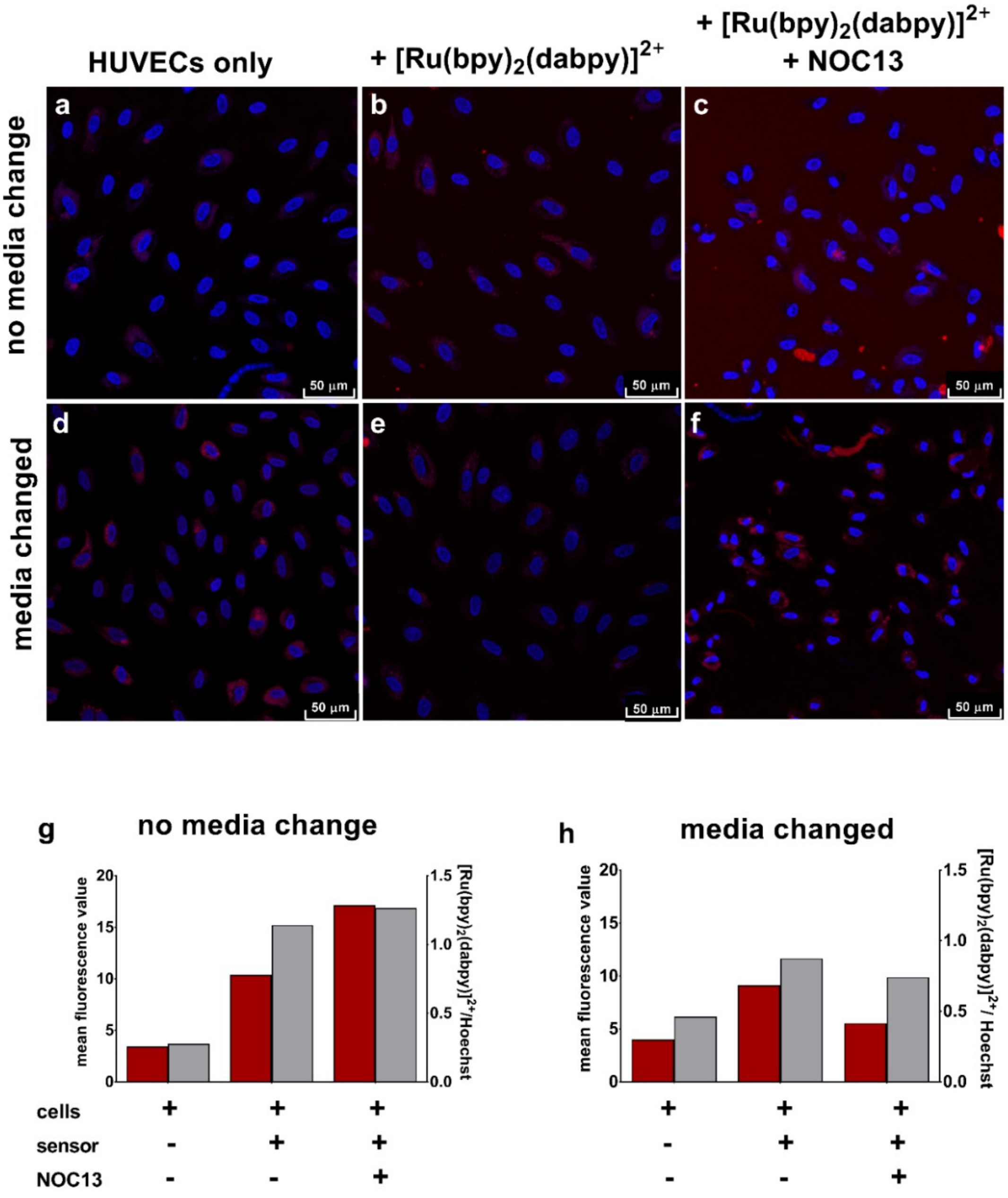

Supplementary Figure S12

Confocal microscopic imaging of HUVECs for exogenous nitric oxide (NO) with NOC13

Representative confocal microscopic images of HUVECs in the presence of the NO sensor $[Ru(bpy)_2(dabpy)]^{2+}$ and (**a** and **c)** 50 µM or (**b** and **d**) 100 µM NOC13 as an exogenous NO donor, in cells before media change.

Red - active/NO bound $[Ru(bpy)_2(T\text{-}bpy)]^{2+}$

Blue - Hoechst 33342 (a nuclear stain)

(**e**) Mean intensities from the red channels in three stacked images are reported (■) alongside the normalised intensities to the nuclear stain (■) in cells subjected to no media change.

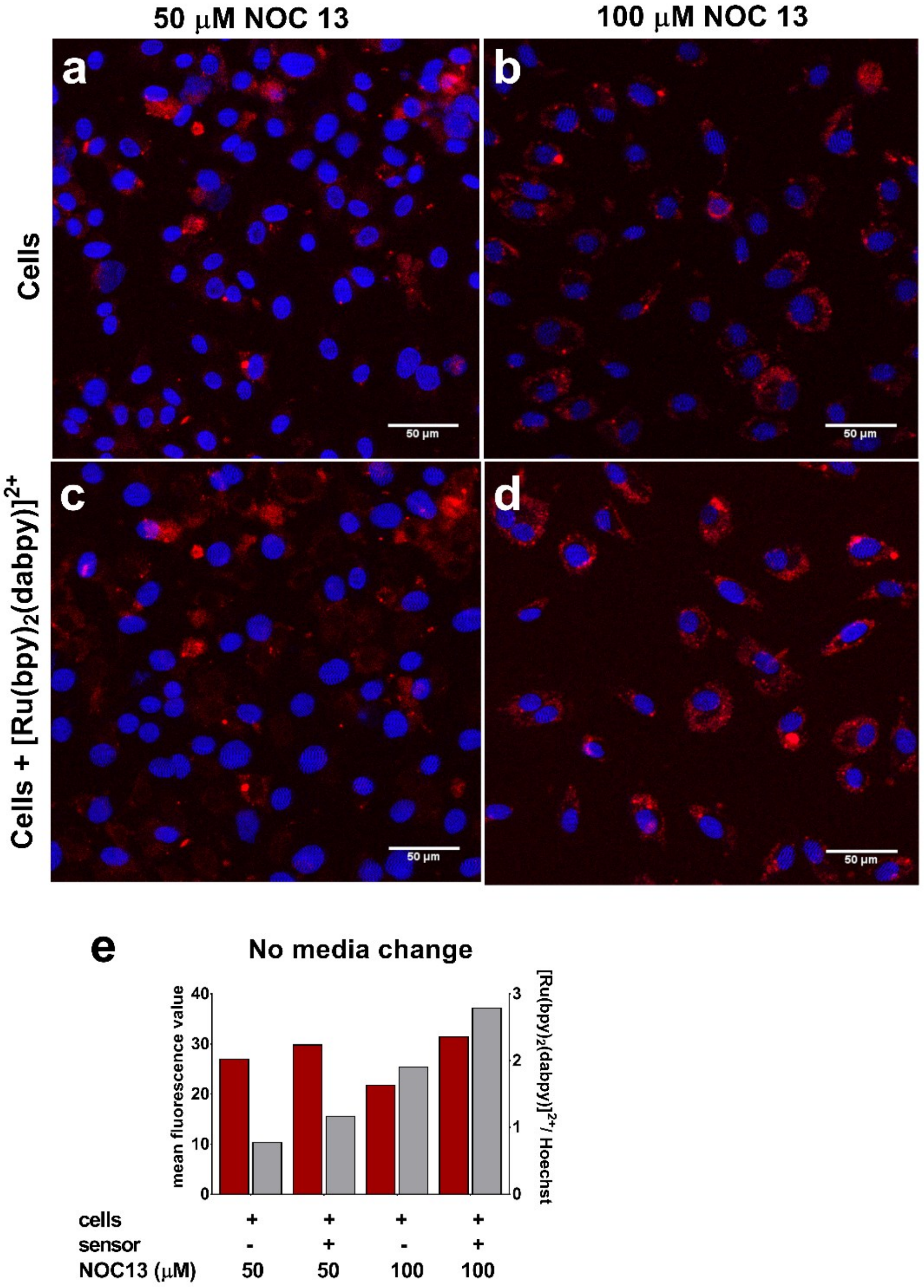

Inductively coupled plasma mass spectrometry (ICP-MS) to assess the cellular uptake of Ruthenium

HUVECs were grown in T75 tissue flasks until 80-90% confluent. Cells were then washed in PBS and trypsinised, harvested and seeded into 6 well plates at 1.2×10^5 cells/well in 2 mL of Meso Endo Cell Growth Medium (Cell Applications INC) and were allowed to adhere to the plates for 24 hours after seeding in the cell incubator (37°C, 5% CO_2). HUVECs were then incubated in triplicate with either PBS, 10 µM or 50 µM $[Ru(bpy)_2(dabpy)]^{2+}$ in Meso Endo media for 24 hours at 37°C, 5% CO_2 in the cell incubator. After 24 hours of incubation with $[Ru(bpy)_2(dabpy)]^{2+}$, the supernatant from each well was removed and placed into a 15 mL tube and spun at 1400 rpm for 3 mins to remove cell debris. HUVECs were washed gently (3 times) with 1 x PBS (1mL), trypsinized and were spun at 7000 rpm for 3 minutes to pellet the cells. The supernatant was removed by suction. Cells were washed by re-suspending in 1 x PBS (1mL) and spun at 7000 rpm for 3 minutes to pellet cells, and repeated 3 times. After the last wash, the cell pellet was desiccated in a dry block heater at 100 °C.

The dry cell samples were then re-suspended in 400 µL of 37% Hydrochloric Acid (HCl, Sigma Aldrich 258148-2.5L) and heated at 100°C until dry (for approximately 2 hours) to digest organic material and liberate Ruthenium from the sample, simplifying the matrix for ICP-MS analysis. The dry samples were then reconstituted in 2% HCl made up in MillQ H_2O (4 mL total volume). All controls as well as the PBS supernatant were diluted 1:20 and the supernatant samples were diluted to fit the calibration series. The 10 µM $[Ru(bpy)_2(dabpy)]^{2+}$ supernatant samples were diluted 1:21.4 and the 50 µM $[Ru(bpy)_2(dabpy)]^{2+}$ supernatant were diluted 1:107.19 in 2% HCl up to a total volume of 4 mL). All samples were sonicated for 20 min to produce a clear solution. The samples were then filtered through a syringe filter cartridge, pore diameter of 0.22 µm (Millex-GV PVDF 0.22 µm, 33 mm) into a 5 mL tube.

The standards were prepared using a 100 mM stock solution of $[Ru(bpy)_2(dabpy)]^{2+}$. Based on the molecular weight of 1071.9, the following dilutions was used to make the standard solutions of 0, 1, 10, 20, 50, 100, 200, 250, 400 and 500 particles per billion (ppb).

ppb	µM	dilution factor
1	0.000933	107190000
10	0.009329	10719000
20	0.018658	5359500
50	0.046646	2143800
100	0.093292	1071900
200	0.186585	535950
250	0.233231	428760
400	0.373169	267975
500	0.466461	214380

Sample solutions were analysed at Adelaide Microscopy, University of Adelaide, with an Agilent 8900x QQQ-ICP-MS. The plasma conditions were: RF power 1550 W, sample depth 8 mm and Ar carrier gas flow rate 1.09 L/min, with a Micro Mist nebuliser and Scott Type spray chamber. The collision cell was run in He mode (4 mL/min He gas flow) for the analysis of ^{99}Ru and ^{101}Ru. On-line addition of In was used as the internal standard element. Calibration solutions at 0, 1, 10, 20, 50, 100, 200, 250, 400, 500 ppb were used for Ru quantification. At the end of the experiments the following standard curves were developed to confirm the accuracy of the standards solutions used to determine the ppb values from the cell lysates and supernatants.

Supplementary Figure S13

Standard curves used for the ICP-MS analysis

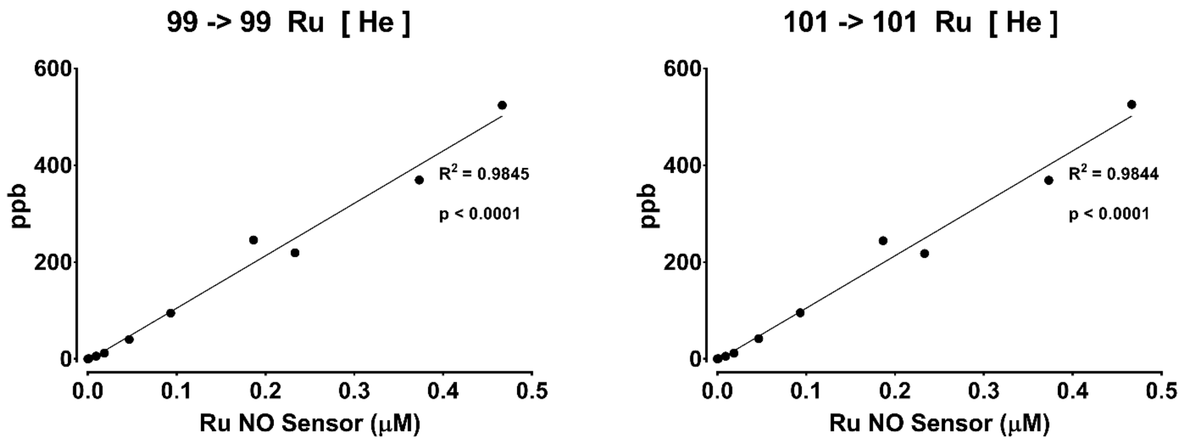

Table S1. ICP-MS analysis of Ruthenium uptake by the HUVECs following exposure to 10 µM or 50 µM NO sensor, $[Ru(bpy)_2(dabpy)]^{2+}$ for 24 hours.

	Sample Name	^{101} Ru Concentration in particles per billion (ppb)	Mean ± s.d. (ppb)	*p* value from One-way ANOVA, Tukey's multiple comparisons test	Percentage in cells [Cell]/[supernatant] X 100%
**Controls**	blank_AM	0.41			
	Meso Endo Media	6.13			
	PBS	2.48			
	Trypsin	2.34			
	2% HCl	1.76			
	DMSO	2.81			
**Supernatant (S/N)**	PBS S/N #1	<0.073			
	PBS S/N #2	<0.073	<0.07		
	PBS S/N #3	<0.073			
	10 µM S/N #1	7321.13	7422.17 ± 102.10		
	10 µM S/N #2	7420.10			
	10 µM S/N #3	7525.31		*p = 0003*	
	50 µM S/N #1	15654.02	18979.59 ± 5355.00		
	50 µM S/N #2	25157.15			
	50 µM S/N #3	16127.63			
**HUVECs (cells)**	PBS cells #1	0.13		*p = 0.04*	
	PBS cells #2	0.08	<0.07		
	PBS cells #3	<0.073			
	10 µM cells #1	2.27	2.40 ± 0.14	*p = 0.001*	
	10 µM cells #2	2.39			**0.032**
	10 µM cells #3	2.55		*p > 0.9*	
	50 µM cells #1	3.84	5.08 ± 1.60		
	50 µM cells #2	4.52			**0.027**
	50 µM cells #3	6.89			

Supplementary Information - Section 3

Western Blot protein analysis of p-eNOS/eNOS

Methods

HUVECs were treated with 0, 5, 10, 25, 50 and 100 µM acetylcholine chloride (Sigma Aldrich)/sterile PBS (phosphate buffered saline) for 15 minutes or 0, 50, 100, 150, 200, 250 and 500 µM of hydrogen peroxide (H_2O_2, Ajax Chemicals)/sterile PBS for 5 minutes. Following these treatments, the HUVECs were lysed and protein extracted using the radioimmunoprecipitation assay (RIPA) buffer (20 mM Tris-HCl, 1 mM EDTA, 1 mM EGTA, 1 mM dithiothreitol, 0.5 mM phenylmethylsulfonyl fluoride, 1.5 µg/ml aprotinin, 1 µg/ml leupeptin, 1 µg/ml pepstatin, 1 mM sodium orthovanadate, and 0.2% Triton X-100; pH 7.4). The protein levels were quantitated using the Pierce bicinchoninic acid (BCA) protein assay kit (Thermo Scientific).

From each condition, 10 µg of protein was run on a 4-12% iBlot mini-gel (Invitrogen) for 1 hr and 20 min at 120 V. Proteins were transferred to PVDF membrane (Invitrogen) using the iBlot transfer stacks system (Invitrogen) and the blot was blocked in 10% (w/v) fat-free milk powder in TBST (Tris-buffered saline with 0.1% Tween 20, Sigma-Aldrich) buffer for 1 hour, at room temperature. The blots were washed with TBST and incubated overnight at 4°C with the primary antibody for p-eNOS/pS1177 (BD Bioscience) at 1:1000 in 2% (w/v) fat-free milk powder in TBST buffer. After washing with TBST, secondary antibody with goat anti-mouse HRP (Santa Cruz Biotechnology) at 1:2000 in 2% (w/v) fat-free milk powder in TBST buffer was added for 2 hours at room temperature. Blots were washed with TBST and incubated with the Clarity Western enhanced chemiluminescence (ECL) Substrate (BIORAD) for 3 min before visualising on the ChemiDoc (BIORAD) to obtain the image for p-eNOS.

The blots were stripped using Stripping Buffer Restore PLUS Western (Thermo Scientific) for 15 min at room temperature, washed in TBST and blocked in 10% (w/v) fat-free milk powder in TBST buffer for 1 hour at room temperature. The blots were washed and incubated overnight at 4°C with primary antibody for eNOS (BD Bioscience) at 1:1000 in 2% (w/v) fat-free milk powder in TBST buffer. After washing the blots with TBST, the secondary antibody with goat anti-mouse HRP (Santa Cruz Biotechnology) at 1:2000 in 2% (w/v) fat-free milk powder in TBST buffer was added for 2 hours at room temperature. Then the blots were washed in TBST and incubated with ECL substrate for 3 min before visualising on the ChemiDoc to obtain the image for total eNOS.

Subsequently, the blot was stripped using Stripping Buffer Restore PLUS Western for 15 min at room temperature, washed in TBST and blocked in 10% (w/v) fat-free milk powder in TBST buffer for 1 hour at room temperature. The blots were washed in TBST and incubated overnight with α-tubulin antibody at 1:5000 (Abcam) in 2% (w/v) fat-free milk powder in TBST buffer. After washing in TBST, the blots were incubated with the ECL substrate for 3 min before visualising on the ChemiDoc to obtain the image for the loading control of α-tubulin. All blots were then quantified and analysed using Image Lab Software 5.0 (BIORAD).

Supplementary Figure S14

Western blots for acetylcholine induced changes in peNOS/eNOS/α-tubulin in HUVECs

The full-length blot images from the same gel, exposure time and the details of the antibodies used with the Clarity Western enhanced chemiluminescence (ECL) substrate for visualising on the ChemiDoc (BIORAD) to obtain the images for **Figure 5.a.**

a. p-eNOS/pS1177 (BD Bioscience) at 1:1000 in 2% (w/v) fat-free milk powder in TBST buffer
Exposure time: 18.308 secs

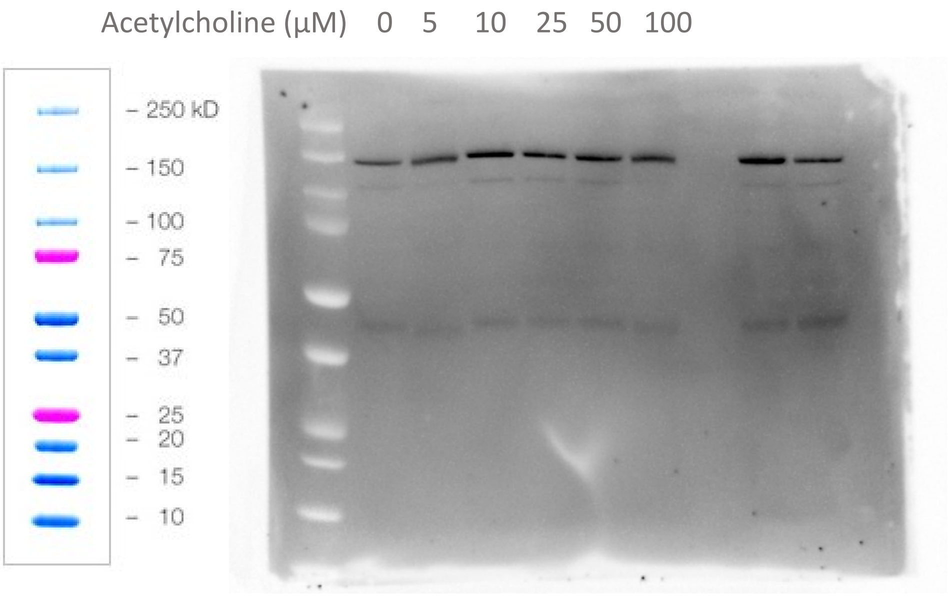

Protein ladder: *BIO-RAD Precision Plus (*peNOS, 140kDa*)*

b. eNOS (BD Bioscience) at 1:1000 in 2% (w/v) fat-free milk powder in TBST buffer
Exposure time: 25.259 secs

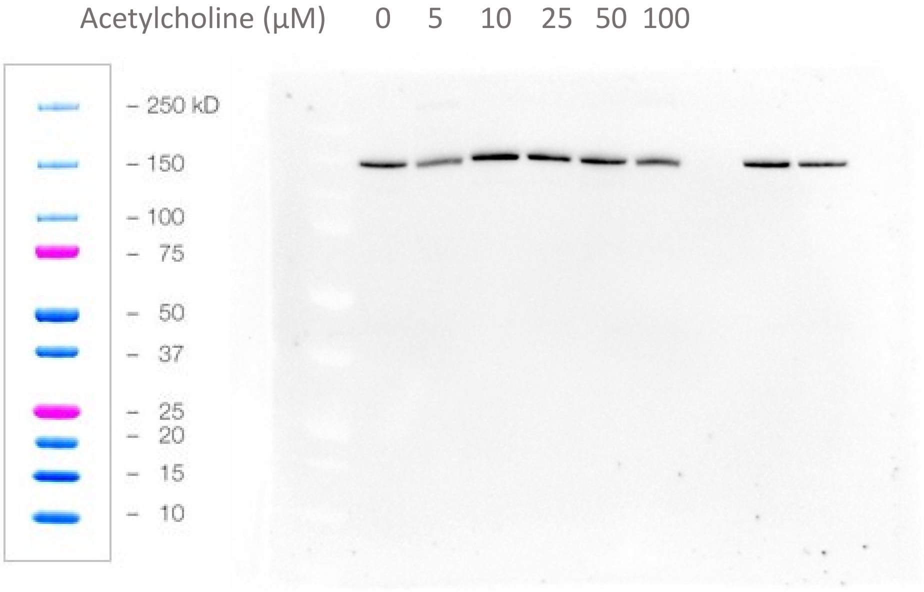

Protein ladder: *BIO-RAD Precision Plus (*eNOS, 140kDa*)*

c. α-tubulin antibody at 1:5000 (Abcam) in 2% (w/v) fat-free milk powder in TBST buffer
Exposure time: 0.243 secs

Acetylcholine (µM) 0 5 10 25 50 100

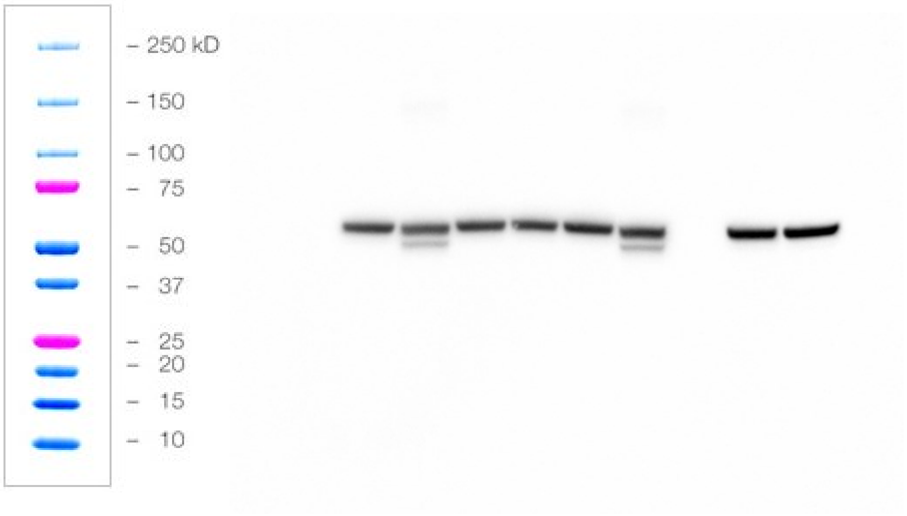

Protein ladder: *BIO-RAD Precision Plus (*α-tubulin, 50kDa*)*

__**Supplementary Figure S15**__

Western blots for hydrogen peroxide induced changes in peNOS/eNOS/α-tubulin in HUVECs

The full-length blot images from the same gel, exposure time and the details of the antibodies used with the Clarity Western enhanced chemiluminescence (ECL) substrate for visualising on the ChemiDoc (BIORAD) to obtain the images for **Figure 6.a**.

a. **p-eNOS/pS1177** (BD Bioscience) at 1:1000 in 2% (w/v) fat-free milk powder in TBST buffer
Exposure time: 10.432 secs

Hydrogen peroxide (µM) 0 50 100 150 200 250 500

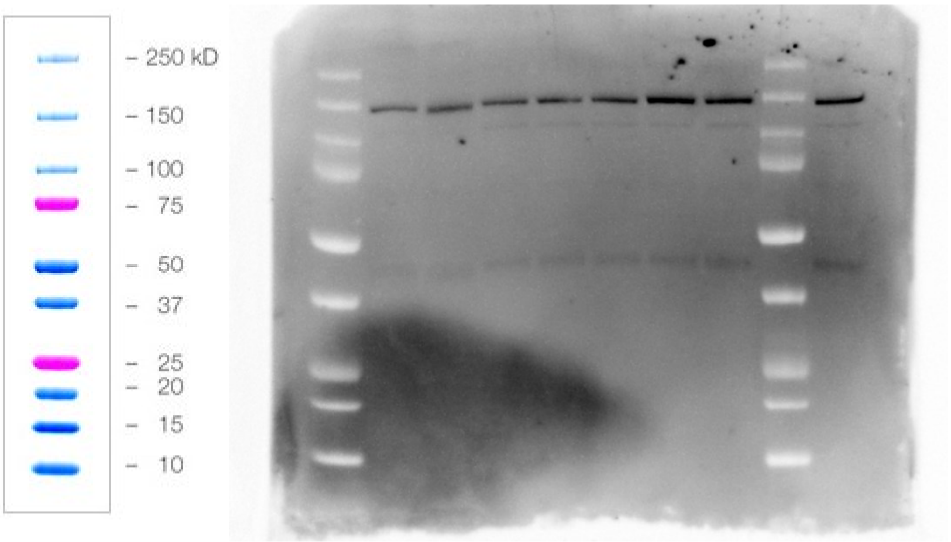

Protein ladder: *BIO-RAD Precision Plus (*peNOS, 140kDa*)*

b. **eNOS** (BD Bioscience) at 1:1000 in 2% (w/v) fat-free milk powder in TBST buffer
Exposure time: 14.865 secs

Hydrogen peroxide (µM) 0 50 100 150 200 250 500

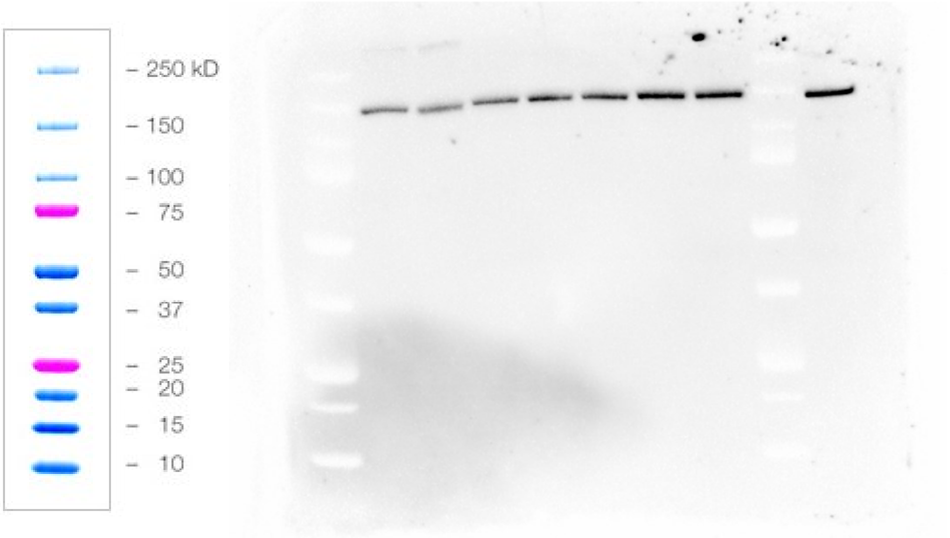

Protein ladder: *BIO-RAD Precision Plus (*eNOS 140kDa*)*

c. **α-tubulin** antibody at 1:5000 (Abcam) in 2% (w/v) fat-free milk powder in TBST buffer
Exposure time: 0.207 secs

Hydrogen peroxide (μM) 0 50 100 150 200 250 500

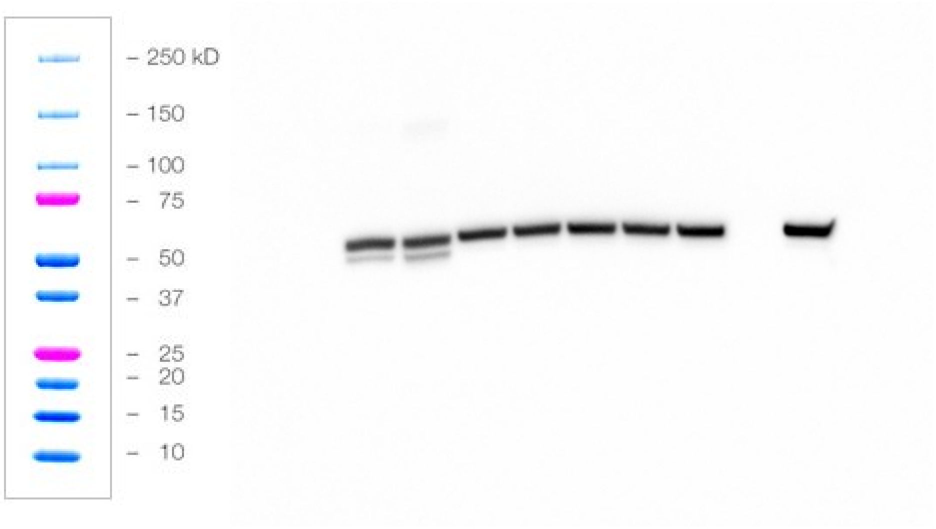

Protein ladder: *BIO-RAD Precision Plus (*α-tubulin, 50kDa*)*

Supplementary Information - Section 4

Detecting NO in plasma using rabbit blood samples

All animal care and handling procedures were approved by Animal Ethics Committee of the South Australian Health and Medical Research Institute (SAHMRI) and the Animal Welfare Committee of the Flinders University, South Australia. All procedures were performed in accordance with the Australian Code for the Care and Use of Animals for Scientific Purposes (2013). New Zealand White, male rabbits were bred in-house at the SAHMRI Preclinical Imaging and Research Laboratory animal facility (Gilles Plains, South Australia) or in the Animal Facility of the Flinders University. Nine rabbits were anaesthetised at two years of age with intramuscular ketamine (35 mg/kg)/xylazine (5 mg/kg) and 2-5% isoflurane inhalation. After thoracotomy, blood was directly drawn from the heart in to a 1 mL syringe containing EDTA/PBS solution and either 500 µL of 100 µM $[Ru(bpy)_2(dabpy)]^{2+}$ in PBS or 500 µL PBS as the vehicle control. The samples were processed in two groups and all readings were done on the SynergyMx Microplate Reader at λ_{ex}=450 nm and λ_{em}=615nm.

Group A (n=4): Thirty minutes after collection (on ice and protected from light), blood samples were centrifuged at 3000 rpm for 10 min, plasma snap-frozen, stored at -80˚C and later thawed prior to reading. Following the initial reading, 50 µL of 1 mM $Ru(bpy)_2(dabpy)]^{2+}$ was added to plasma from the negative control (containing only PBS with plasma) and re-read to determine the changes of NO levels in plasma over time after a freeze-thaw cycle. NOC13 (1 mM) was then added to all samples containing $[Ru(bpy)_2(dabpy)]^{2+}$ and re-read after 10 minutes to confirm the presence of active $Ru(bpy)_2(dabpy)]^{2+}$ in plasma after sample processing.

Supplementary Figure S16

Scheme for sample processing in Group A rabbits.

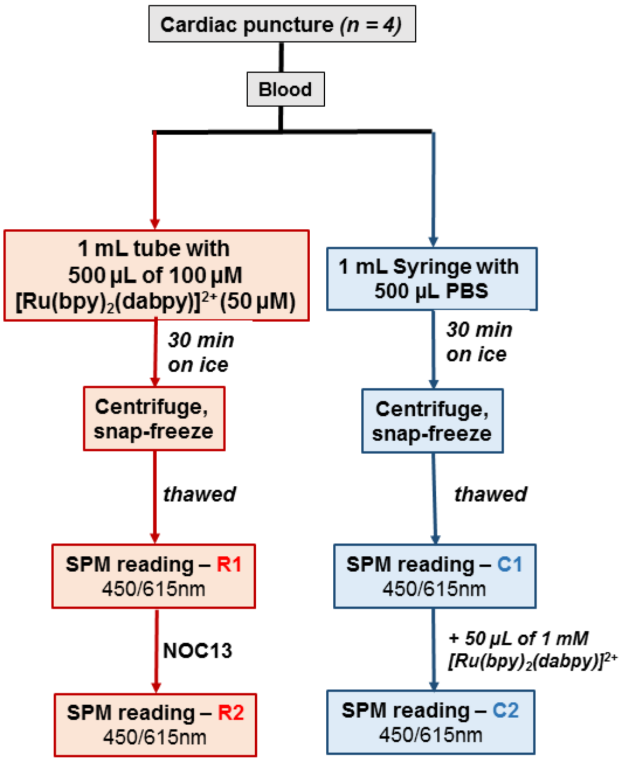

SPM = Spectrophotometer

Figure **8.a**: C1 vs R1

Figure **8.c**: R1 vs C2

Figure **8.e**: R1 vs R2

Group B (n=5): Both samples of blood were left on ice for 20 min covered from light. Then, 50 µL of 1 mM $Ru(bpy)_2(dabpy)]^{2+}$ was added to the negative control containing PBS and left for 10 min. 50 µL of PBS was added to the other sample to control for the volume. All samples were centrifuged 3000 rpm 10 min, plasma separated, snap frozen, stored at -80˚C and thawed prior to reading. Following the initial read, the plate was left in -80˚C for 48 hours and re-read to determine the stability of NO-$[Ru(bpy)_2(dabpy)]^{2+}$ complex over time and following freezing.

<u>Supplementary Figure S17</u>

Scheme for sample processing in Group B rabbits.

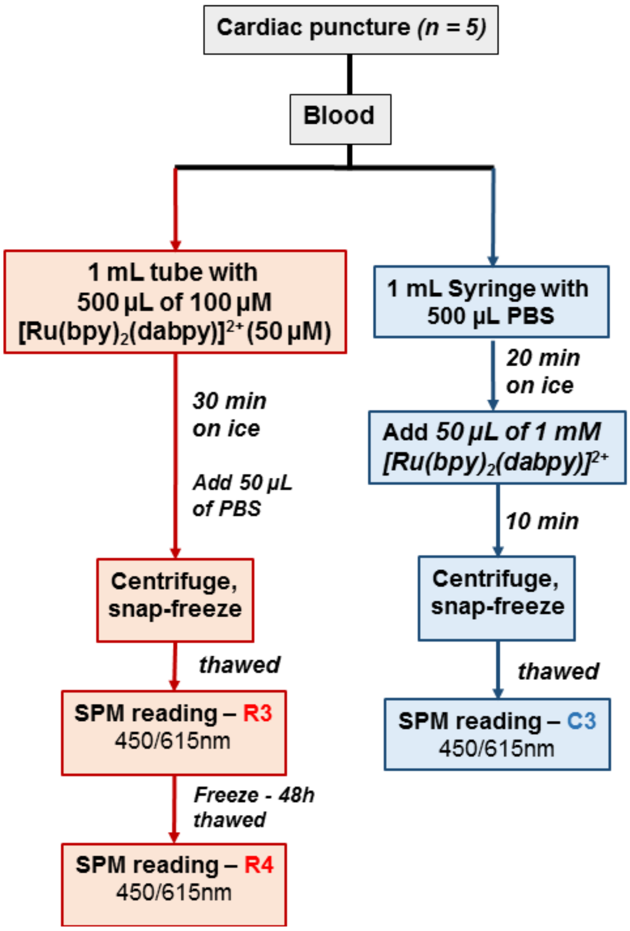

SPM = Spectrophotometer

Figure **8.b**: R3 vs C3

Figure **8.d**: R3 vs R4

In addition, blood from four rabbits (3-4 years old) were used for control experiments with and without a scavenger for NO.

Group C (n=4): Two samples of blood were collected to tubes containing either $[Ru(bpy)_2(dabpy)]^{2+}$ alone or with cPTIO (200 µM). Both samples were centrifuged 3000 rpm 10 min, plasma separated, snap frozen, stored at -80˚C and thawed prior to reading.

<u>Supplementary figure S18</u>

Scheme for sample processing in Group C rabbits.

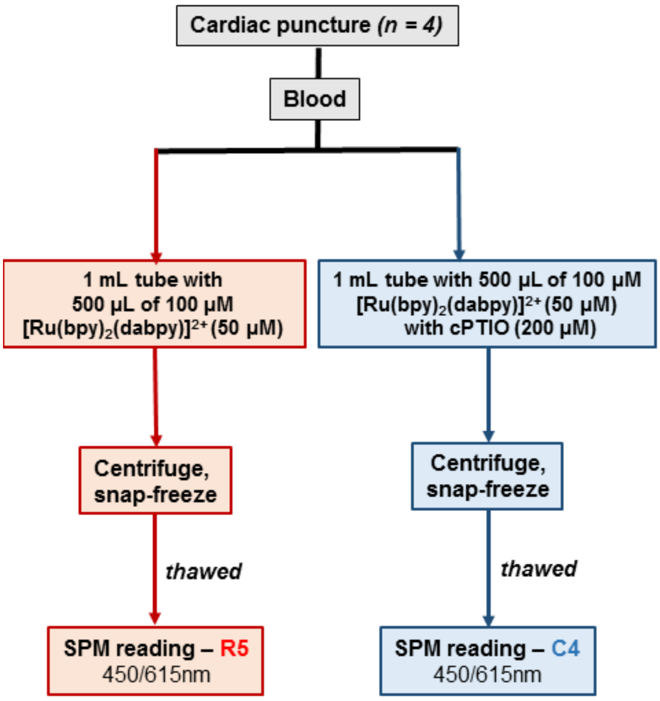

SPM = Spectrophotometer

Figure **8.f**: R5 vs C4
